# The bZIP Transcription Factor Rca1p Is a Central Regulator of a Novel CO_2_ Sensing Pathway in Yeast

**DOI:** 10.1371/journal.ppat.1002485

**Published:** 2012-01-12

**Authors:** Fabien Cottier, Martine Raymond, Oliver Kurzai, Marianne Bolstad, Worraanong Leewattanapasuk, Claudia Jiménez-López, Michael C. Lorenz, Dominique Sanglard, Libuše Váchová, Norman Pavelka, Zdena Palková, Fritz A. Mühlschlegel

**Affiliations:** 1 School of Biosciences, University of Kent, Canterbury, United Kingdom; 2 Singapore Immunology Network, Agency for Science, Technology and Research, Immunos, Singapore; 3 Institute for Research in Immunology and Cancer and Department of Biochemistry, Université de Montréal, Montréal, Quebec, Canada; 4 Septomics Research Centre, Friedrich-Schiller-University and Leibniz-Institute for Natural Products Research and Infection Biology, Hans-Knoell-Institute, Jena, Germany; 5 Department of Microbiology and Molecular Genetics, University of Texas Health Science Center, Houston, Texas, United States of America; 6 Institute of Microbiology, University of Lausanne and University Hospital Center, Lausanne, Switzerland; 7 Institute of Microbiology of the ASCR, v.v.i., Prague, Czech Republic; 8 Department of Genetics and Microbiology, Faculty of Science, Charles University, Prague, Czech Republic; 9 Clinical Microbiology Service, East Kent Hospitals University NHS Foundation Trust, Ashford, United Kingdom; University of Toronto, Canada

## Abstract

Like many organisms the fungal pathogen *Candida albicans* senses changes in the environmental CO_2_ concentration. This response involves two major proteins: adenylyl cyclase and carbonic anhydrase (CA). Here, we demonstrate that CA expression is tightly controlled by the availability of CO_2_ and identify the bZIP transcription factor Rca1p as the first CO_2_ regulator of CA expression in yeast. We show that Rca1p upregulates CA expression during contact with mammalian phagocytes and demonstrate that serine 124 is critical for Rca1p signaling, which occurs independently of adenylyl cyclase. ChIP-chip analysis and the identification of Rca1p orthologs in the model yeast *Saccharomyces cerevisiae* (Cst6p) point to the broad significance of this novel pathway in fungi. By using advanced microscopy we visualize for the first time the impact of CO_2_ build-up on gene expression in entire fungal populations with an exceptional level of detail. Our results present the bZIP protein Rca1p as the first fungal regulator of carbonic anhydrase, and reveal the existence of an adenylyl cyclase independent CO_2_ sensing pathway in yeast. Rca1p appears to regulate cellular metabolism in response to CO_2_ availability in environments as diverse as the phagosome, yeast communities or liquid culture.

## Author Summary

Skin infection, oral and vaginal thrush, or bloodstream candidiasis are some of the diseases caused by the human pathogen *Candida albicans*. The high versatility of infection niches reflects the capacity of this yeast to respond to strong variations in its environment such as CO_2_ concentration. This molecule initiates the regulation of an essential protein: carbonic anhydrase, not through the known adenylyl cyclase CO_2_ sensor but as we discovered via a novel fungal CO_2_ sensing pathway involving the transcriptional regulator Rca1p. This protein is additionally implicated in growth, yeast-to-hyphae morphological switch and cell wall stability of *C. albicans*. The ortholog of Rca1p in *Saccharomyces cerevisiae* demonstrated a conserved function in the induction of the carbonic anhydrase in low CO_2_ concentration atmospheres pointing to the broad significance of Rca1p in fungal CO_2_ sensing.

## Introduction

Atmospheric carbon dioxide (CO_2_) with a concentration of 0.039% is not only central to the Earth's biogeochemical carbon cycle but is also sensed as a signal by many organisms. The nematode and parasite of insects *Neoaplectana carpocapsae* localizes its prey via a CO_2_ gradient [1], while avoidance behaviour in another nematode, *Caenorhabditis elegans* [2], or the model organism *Drosophila melanogaster* is provoked by elevated CO_2_ [3]. *C. elegans* detects CO_2_ via a cGMP-gated ion channel [2] whereas in *D. melanogaster* CO_2_ is sensed by a pair of 7 transmembrane domains chemoreceptors localized on specialized sensory neurons [4].

In the fungal kingdom CO_2_, under its hydrated form bicarbonate (HCO_3_^−^), is critical for cellular metabolism. Although hydration of CO_2_ to HCO_3_^−^ and a proton occurs spontaneously, this reaction is greatly enhanced by the metalloenzyme Carbonic Anhydrase (CA), which operates at a rate of up to 10^6^ reactions per second [5]. Fungal CAs fix the membrane permeable gas CO_2_ as HCO_3_^−^ inside the cell, which is subsequently used as substrate for fundamental carboxylation reactions including the conversion of acetyl-CoA to malonyl-CoA (EC 6.4.1.2), or pyruvate to oxaloacetate (EC 6.4.1.1). The direct relevance of HCO_3_^−^ synthesis for fungal survival is reflected by the fact that the CA deletion mutants of *Candida albicans*, *Cryptococcus neoformans*, *Saccharomyces cerevisiae*, *Sordaria macrospora*, *Aspergillus fumigatus* or *Aspergillus nidulans* fail to grow in ambient air [6], [7], [8], [9], [10]. However, when cultured in a CO_2_^−^enriched atmosphere, where sufficient HCO_3_^−^ is spontaneously formed to meet the metabolic requirements, CAs are optional.

In fungi CO_2_ is also sensed as a signal to regulate the expression of virulence factors. In the pathogenic yeast *C. albicans*, high level of CO_2_ triggers filamentous growth and the white-opaque switch [7], [11]. Recently we have shown that in *C. albicans* CO_2_/HCO_3_^−^ is detected by the enzyme adenylyl cyclase Cyr1p which regulates most processes considered essential in *C. albicans* virulence [7], [12]. Here, Cyr1p senses CO_2_/HCO_3_^−^ by a lysine residue (position 1373) of the C-terminal catalytic-site [13] potentially linking HCO_3_^−^, generated by CA, and cAMP signaling. In humans CAs are involved in medically relevant processes including bone calcification, or renal clear-cell-carcinoma progression; consequently, understanding their regulation and use of inhibitors has attracted considerable interest [14]. This led to the identification of the first regulator of CA, the bHLH transcription factor HIF-1α, which controls the expression of major hypoxia-induced genes including CA IX [15]. Another recently identified CA regulator is AphB from *Vibrio cholera* [16]. This LysR-type transcription factor also activates the ToxR virulence cascade via the *tcpPH* operon which ultimately induces the production of cholera toxin.

Notably the CAs of *S. cerevisiae* [17], [18], *S. macrospora* [8], *A. fumigatus* and *A. nidulans* [9] are expressed in response to the availability of environmental CO_2_. However, fungal genomes do not posses orthologs of either HIF-1α or AphB-type CA regulators. This suggests the existence of an, as yet, undiscovered CO_2_ signaling mechanism controlling fungal CA expression.

In this report we investigate the existence of such a pathway in fungi by using, as a model, the well characterized CO_2_ sensing system of the pathogenic yeast *C. albicans*. We have shown that *C. albicans* posses a single β-CA, required for growth under CO_2_ limiting atmosphere [7]. We now demonstrate that the expression of both transcript and protein of this CA is controlled by the level of environmental CO_2_ and that CA is further induced in an *ex vivo* model of phagocytosis by mammalian phagocytes, suggesting that CO_2_ might be limiting even in the relatively high CO_2_ conditions in the host. We find that such regulation in *C. albicans* is independent from the already known sensor adenylyl cyclase, described above, suggesting the existence of a cAMP-independent CO_2_ signaling pathway in fungi. By implementing a systematic functional screen we identify the bZIP transcription factor Rca1p as the *C. albicans* regulator of CA expression in response to CO_2_ availability. Furthermore, by using Chromatin Immuno Precipitation (ChIP) on chip and ChIP-qPCR experiments we confirm that Rca1p binds to the CA promoter, and to 84 additional genes. The broad significance of our findings is further underlined by our data revealing the existence of a conserved CO_2_ sensing pathway controlled by an Rca1p ortholog in the model organism *S. cerevisiae*. Finally, using advanced microscopy, we contribute to the understanding of CO_2_ flux and metabolic adaptation inside yeast populations on a hitherto unprecedented level of resolution.

## Results

### Yeast carbonic anhydrases are expressed according to CO_2_ availability

**Figure 1:**
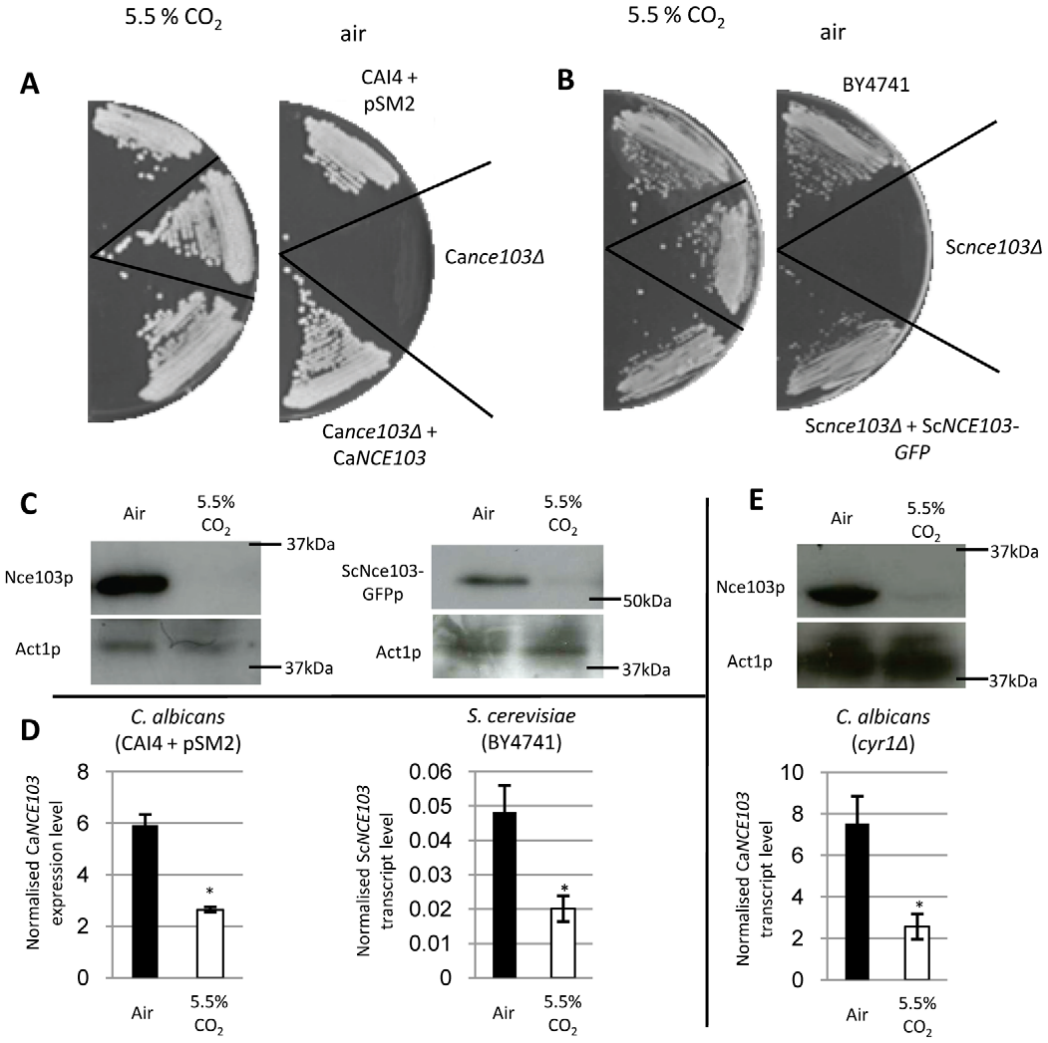
*NCE103* is essential for growth of *C. albicans* and *S. cerevisiae* and its expression is controlled by the concentration of environmental CO_2_. Inactivation of the β-carbonic anhydrase encoded by *NCE103* in (**A)** *C. albicans,* (**B**) *S. cerevisiae* inhibits growth in ambient air (right set of pictures) but not in air enriched with 5.5% CO_2_ (left set of pictures). All strains were incubated on YPD medium for 24 hours. **C**) Western blots from *C. albicans* (left) and *S. cerevisiae* (right). Yeast carbonic anhydase is present in higher quantity in air than air enriched with 5.5% CO_2_ samples. **D**) qRT-PCR using *NCE103* specific primers and RNA extracted from *C. albicans* (left) and *S. cerevisiae* (right) grown in air (black columns) or air enriched with 5.5% CO_2_ (white columns). **E**) Western blot (top) and qRT-PCR (bottom) relative to *C. albicans cyr1*Δ. Data are represented as mean +/− SD. Asterisk indicates statistical significance determined by two-sample *t* test (*P*≤0.05).

The CA, Nce103p, from *C. albicans* and *S. cerevisiae* are known to be required for growth in ambient air ([6], [7] and Figure 1A, B). However, to allow an in-depth analysis of yeast CA expression we developed an antibody directed against *C. albicans* Nce103p, and additionally constructed a strain expressing a functional tagged CA in *S. cerevisiae*: Sc*nce103*Δ+Sc*NCE103-GFP* (Figure 1B).

Since the CAs from *C. albicans* and *S. cerevisiae* are required for growth in a low but not high CO_2_ atmosphere we asked whether expression of the enzyme itself is regulated by the availability of this gas. To address this question we performed Western-blot analysis using appropriate antibodies to detect *C. albicans* CA and CA chimera of *S. cerevisiae*. Single bands with the predicted molecular weights for CaNce103p (32kDa), and ScNce103-GFPp (54kDa) were detected and we now show for the first time that CA protein expression is highly regulated in both yeast species (Figure 1C). In fact, CA is strongly expressed when yeasts are grown in ambient air but non-detectable when cultured in air enriched with 5.5% CO_2_, precisely mirroring the requirements for growth of the *NCE103* gene in yeast (Figure 1A, B). Furthermore, quantitative real time polymerase chain reaction (qRT-PCR) analysis with reverse transcripts of total RNA extracted from *C. albicans* and *S. cerevisiae* grown at low and high CO_2_ concentrations show a similar regulation of CA transcript when expressions were normalized to *ACT1* (Figure 1D), confirming previous reports made in *S. cerevisiae* [17], [18].

### CO_2_ regulation of carbonic anhydrases in yeast is independent of cAMP signaling

We have previously shown that the adenylyl cyclase Cyr1p from *C. albicans* functions as a major CO_2_-sensor, promoting the yeast-to-hyphae switch in response to high levels of CO_2_ [7], [13]. We now asked if CO_2_ regulation of CA expression was similarly coordinated by Cyr1p or cAMP signaling examining CA protein and transcript levels in a strain where both alleles of *CYR1* have been deleted (*cyr1Δ*). Notably, CO_2_ regulation of both protein and transcript levels of *NCE103* remain unaltered in the *cyr1Δ* strain displaying a pattern of expression identical to the control strain CAI4+pSM2 (Figure 1E). Furthermore, Western-blot or qRT-PCR analysis revealed that supplementation of culture media with exogenous cAMP at concentrations known to mimic Cyr1p activity [19] (10 mM) did not affect CA expression in CAI4+pSM2 grown in ambient air (Figure S1). Similarly to *C. albicans*, addition to the growth media of 10 mM cAMP did not affect the expression of CA protein or transcript in *S. cerevisiae* (Figure S1). Taken together our data demonstrate that CO_2_ regulation of CA in *C. albicans* is independent of the known CO_2_ sensor Cyr1p and its product cAMP. Furthermore, they strongly support the existence of a novel, cAMP-independent, CO_2_ signaling pathway in yeast.

### The bZIP transcription factor Rca1p is a new regulator of CO_2_ signaling in *C. albicans*

**Figure 2:**
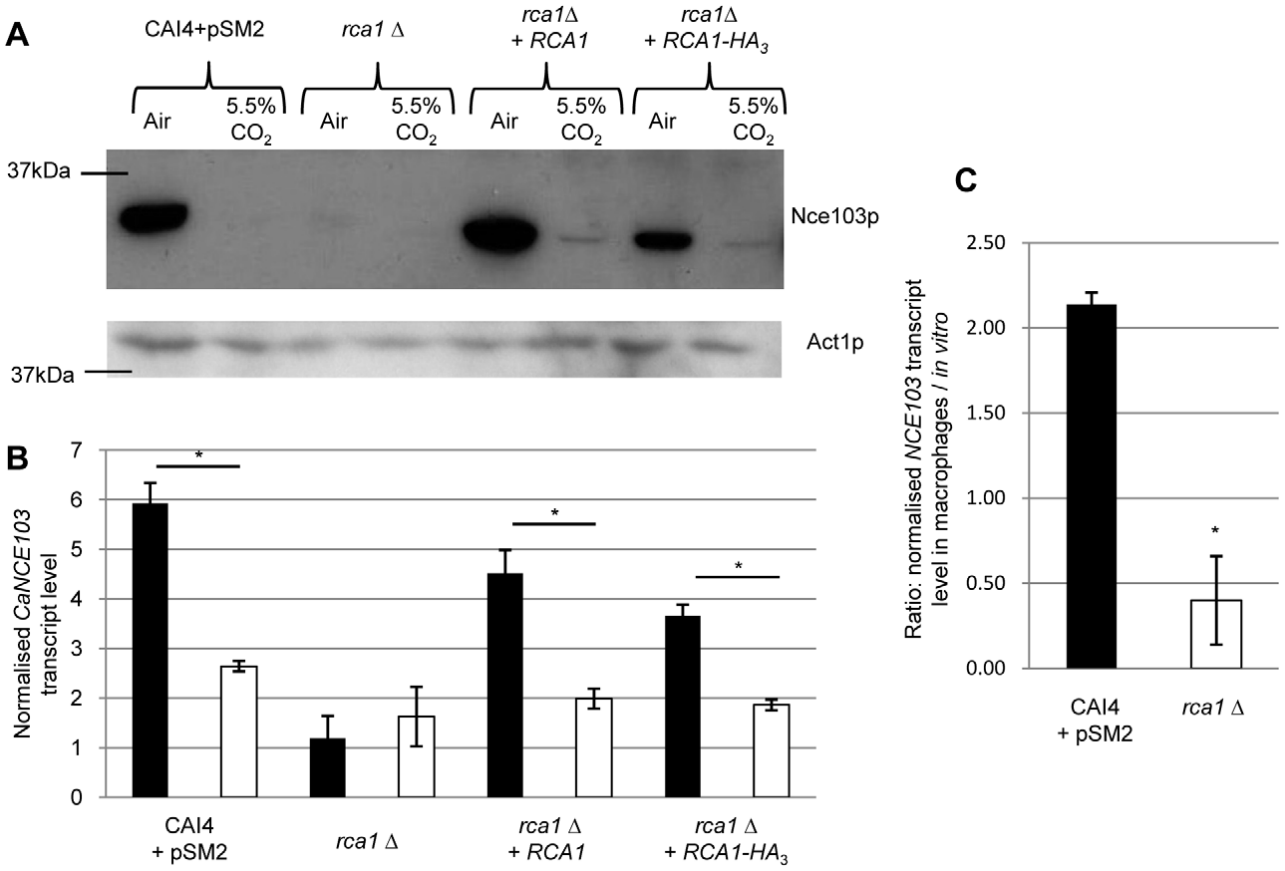
The bZIP transcription factor Rca1p is a regulator of CO_2_ signaling in *C. albicans*. **A**) Western blots with protein extract from the *C. albicans* control strain, *rca1*Δ and *rca1* complemented strains. **B**) qRT-PCR using *NCE103* specific primers and RNA extracted from the above strains grown in air (black columns) or air enriched with 5.5% CO_2_ (white columns). **C**) qRT-PCR with *NCE103* specific primers were used to calculate the ratio of transcript between cells phagocyted and cells grown *in vitro* in the control (black column) or *rca1*Δ strains (white column). Data are represented as mean +/− SD. Asterisk indicates statistical significance determined by two-sample *t* test (*P*≤0.05).

To identify the key components of this novel CO_2_ sensing pathway we systematically screened a *C. albicans* knock-out library searching for strains with an altered expression of their CA in response to CO_2_. The library consisted of 158 *C. albicans* non-essential transcription factor mutants (provided by D. Sanglard). CA protein expression was investigated in each mutant grown in either ambient air or air enriched with 5.5% CO_2_. Repeated screening identified a single candidate (HZY7-1) that failed to induce CA protein when grown in ambient air. HZY7-1 harbors a mutation in the *C. albicans orf19.6102* gene. To confirm the HZY7-1 phenotype, we independently inactivated the two *orf19.6102* alleles in a CAI4 background, using the URA-blaster approach [20], and re-introduced *URA3* at its native locus to generate strain *rca1*Δ. Subsequent to validating gene inactivation by Southern blot and qRT-PCR (Figure S2 and S3), we confirmed a striking loss of Nce103p protein induction in *rca1*Δ in ambient air (Figure 2A). We also validated that during the inactivation process, we did not alter the expression of 2 genes partially overlapped by *RCA1*: *orf19.6103* and *MVD* (Figure S3). In light of these findings we named the gene encoded by *orf19.6102*: *Regulator of Carbonic Anhydrase 1. RCA1* encodes a 283 amino acid (aa) hypothetical protein which contains a conserved basic leucine zipper (bZIP) domain in its C-terminus, required for DNA interaction (Figure S4). Reintroduction of *RCA1,* either on its own (*rca1*Δ+*RCA1*) or tagged at its C-terminus with Haemagglutinin (*rca1*Δ+*RCA1*−*HA_3_*), into the *rca1*Δ strain restored CA protein induction in *C. albicans* cells exposed to low CO_2_ level (Figure 2A). These observations were also confirmed by qRT-PCR (Figure 2B).

### *C. albicans NCE103* induction in an *ex vivo* virulence model is dependent on Rca1p

In a previous study of transcriptional variation that follow phagocytosis of *C. albicans* by murine macrophages, *NCE103* was found to be mildly induced (∼1.9-fold after two hours of co-culture [21], while in *S. cerevisiae, NCE103* was one of the genes most highly induced by phagocytosis (13.8-fold; [22]). We assayed expression of *CaNCE103* in phagocytosed *C. albicans* cells after one hour of co-culture by qRT-PCR and found an induction of 2.1-fold relative to cells in media alone, even though both populations were exposed to a high-CO_2_ environment (5.0% in a tissue culture incubator). This change is of similar magnitude, but slightly faster, than observed by microarray. This induction was completely absent in an *rca1*Δ strain (Figure 2C). These results indicate that Rca1p regulates Ca*NCE103* in a physiological environment which could be correlated to a CO_2_ concentration scarcer within the immune cell due to a limited penetration across multiple membranes, the sequestering activity of the mammalian CAs, or the reduced metabolic production of CO_2_ in the fungal cell as a result of a shift to slower, and gluconeogenic growth.

### CO_2_ regulation of carbonic anhydrases by Rca1p orthologs is conserved in *S. cerevisiae*

**Figure 3:**
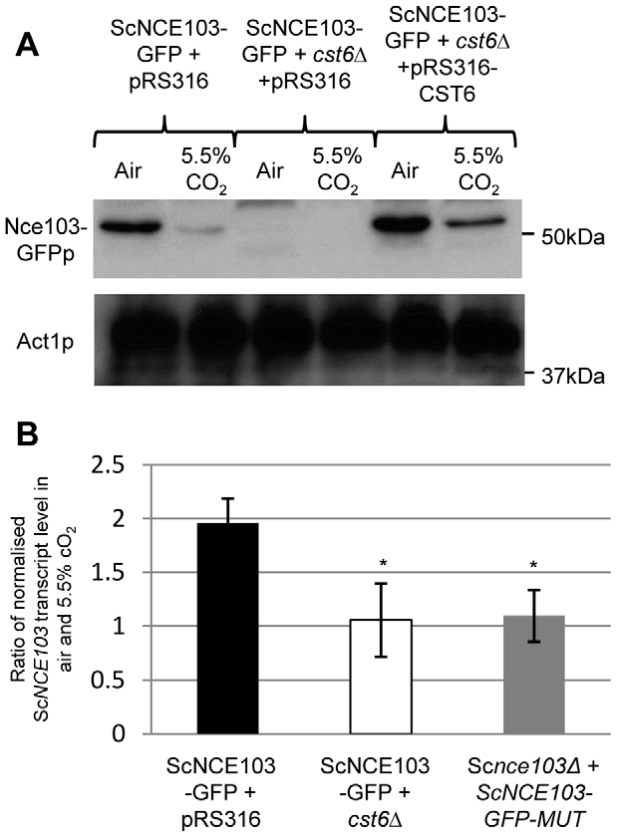
Rca1p orthologs regulate *NCE103* expression in *S. cerevisiae* via a TGACGTCA binding motif. **A**) Western blot with extracts from *S. cerevisiae Sc*NCE103−GFP+pRS316 control strain, *cst6*Δ mutant and the complemented strain (ScNCE103−GFP+*cst6*Δ+pRS316−CST6). **B**) qRT-PCR with specific primers were used to calculate the ratio of *NCE103* transcript between low (air) and high CO_2_ (5.5%) in *S. cerevisiae* control strain (black column), *cst6*Δ mutant (white column) and point mutation in the promoter of *NCE103* (Sc*nce103Δ*+*ScNCE103*−*GFP*−*MUT*) (grey column). Data are represented as mean +/− SD. Asterisk indicates statistical significance determined by two-sample *t* test (*P*≤0.05).

Since CA expression in *S. cerevisiae* is also controlled by ambient CO_2_ levels we investigated the existence of Rca1p orthologs in this yeast. In *S. cerevisiae*, we identified Cst6p (BLAST; Score: 117; E value: 1e^−16^) as a potential Rca1p ortholog. Cst6p encodes for a putative 587 aa protein with a bZIP domain in the C-terminus (Figure S4). In order to prove that Cst6p is a yeast CA regulator we constructed the mutants in the *S. cerevisiae* ScNCE103-GFP background (ScNCE103−GFP+*cst6Δ*). Successful gene inactivations were confirmed by diagnostic PCR and qRT-PCR (Figure S2 and S3). Using anti-GFP antibodies for ScNCE103−GFP+*cst6Δ* we found that its CA, similar to the *C. albicans rca1Δ* strain (Figure 2A), was not induced in low ambient CO_2_ when compared to the controls (Figure 3A). This regulation in *S. cerevisiae* mutant was also confirmed at transcript level by qRT-PCR (Figure 3B). In the mutant, introduction of a plasmid expressing *CST6* restores the expression of *NCE103* in air (Figure 3A). Taken together these data show that CO_2_ regulation by Rca1p orthologs is conserved in yeast.

### CO_2_ regulation of *NCE103* in *S. cerevisiae* occurs through a specific DNA binding motif

*S. cerevisiae* Cst6p is a transcription factor previously described to bind a specific DNA motif: TGACGTCA [23]. We identified this motif in the *NCE103* promoters of *S. cerevisiae* (position -285 bp to ATG), but not of *C. albicans*. To assess the role of this motif in CO_2_ regulation of CA expression we neutralized it by removing 7 and 4 bases pairs of the TGACGTCA sequence in the promoters controlling Nce103p expression in *S. cerevisiae*. Notably the resulting strains (Sc*nce103*Δ+Sc*NCE103*−*GFP*−*MUT*) failed to induce CA when exposed to low environmental CO_2_ (Figure 3B), exactly mirroring the expression pattern displayed by the *S. cerevisiae cst6Δ* mutants (Figure 3B). In summary, our data show that CO_2_ regulation of CAs expression in yeast is controlled by a conserved transcriptional factor, but involves divergent DNA motifs between *S. cerevisiae* and *C. albicans*.

### ChIP-chip analysis of Rca1p confirms *NCE103* binding and points to a wider role in *C. albicans* CO_2_ sensing

**Figure 4:**
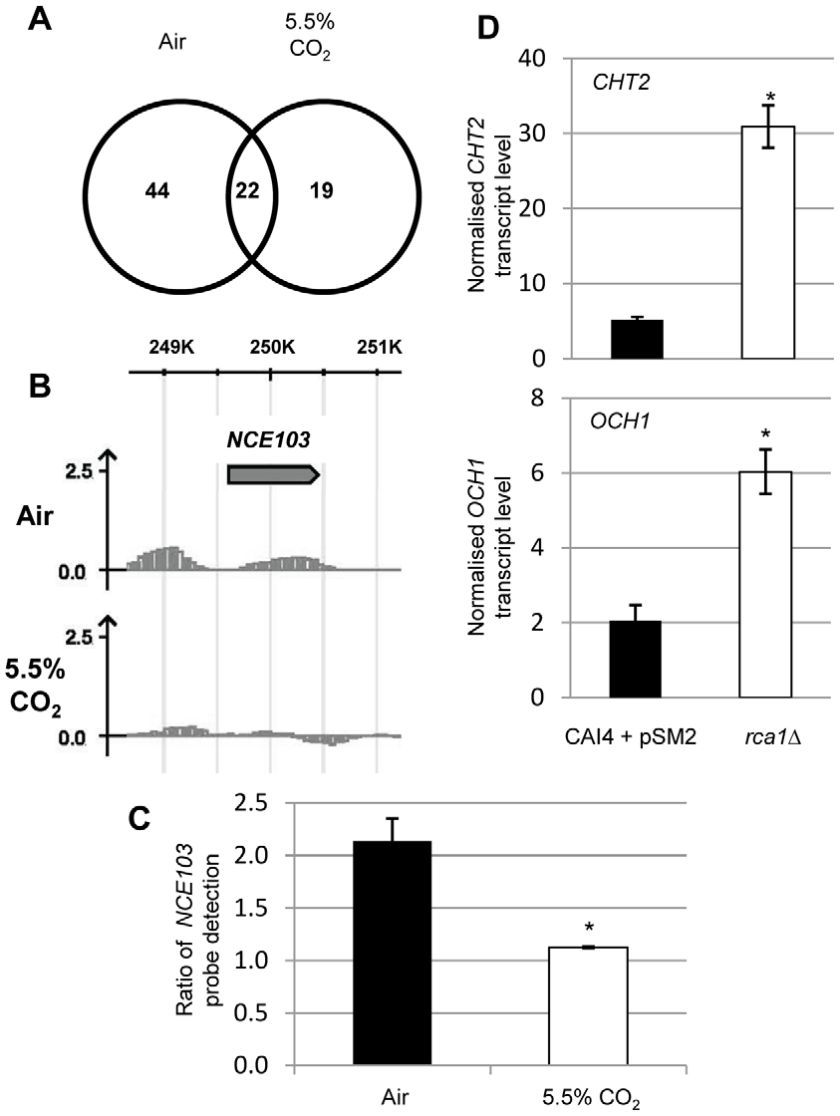
Rca1p is associated to *NCE103* and cell wall structure genes. **A**) Venn diagram of the Rca1p associated genes. **B**) Rca1p binds to the *NCE103* promoter. Representation of the normalized log_2_-transformed signal intensities of RCA1-HA_3_-tagged in air (top panel) and high CO_2_ (bottom panel) compared to the untagged strain versus the corresponding position of each signal on *C. albicans* genomic regions. Log_2_-transformed signal intensity values are indicated at the left of the *y*-axis, the reference is the value 0 (i.e., a binding ratio of 1). **C)** ChIP-qPCR of RCA1-HA_3_ tagged strain versus untagged control in air and a 5.5% CO_2_ environment normalized to *ACT1* level with primers designed to amplify the above identified binding region of Rca1p on the *NCE103* promoter. **D**) qRT-PCR carried out with primers for *C. albicans CHT2* (top) and *OCH1* (bottom) on total RNA extracted from the *C. albicans* control strain (black columns) and *rca1*Δ (white columns) grown in air. Data are represented as mean +/− SD. Asterisk indicates statistical significance determined by two-sample *t* test (*P*≤0.05).

To confirm that Rca1p directly binds to the CA promoter, and identify any additional genes it controls, we performed Chromatin Immuno Precipitation on Chip (ChIP-Chip) in air and air enriched with 5.5% CO_2_. We introduced the HA-tagged *RCA1* allele, described above, into the heterozygous *RCA1* mutant (*rca1*Δ/*RCA1*). The resulting strain (*rca1*Δ/*RCA1*+*RCA1*−*HA_3_*) expressed one wild-type and the HA-tagged *RCA1* copy. Next, we confirmed that CA levels in *rca1*Δ/*RCA1*+*RCA1*−*HA_3_* and in control strain *rca1*Δ/*RCA1*+*RCA1* were fully responsive to CO_2_ by Western blotting, using anti-Nce103 antibodies (Figure S5). Subsequently, genome-wide location profiling of Rca1-HA_3_p in low and high CO_2_ using *C. albicans* whole-genome oligonucleotide tiling arrays [24] produced a total of 182 binding peaks, when the experiment was carried out in air, and 140 in air enriched with 5.5% CO_2_ (log2-transformed pseudomedian signal intensity cutoff: 0.5; P≤0.01) (Table S1 and S2) including 61 common “hits” between the two conditions. In depth analysis revealed that 85 of the hits could directly be associated with ORFs (hits located within 1kbp before an ATG start codon) (Figure 4A). Notably, we identified significant enrichments of several consecutive probes localized in the promoter of *NCE103* (between position −654 and −479bp before ATG) in samples extracted from cells grown in air but not in those supplemented with 5.5% CO_2_ (Figure 4B). In addition to the promoter region, Rca1p binding was also enriched in the coding region of *NCE103* (Figure 4B). This binding profile has been previously reported for another bZIP transcriptional factor, Cap1p, and suggests binding of the protein to the transcriptional machinery [25]. To confirm the association of Rca1p to the CA promoter, we performed ChIP in tagged and untagged strains grown in low and high CO_2_, followed by a qPCR with primers specifically designed to amplify the predicted binding region of Rca1p on the *NCE103* promoter. As expected, we observed a 2.13 fold enrichment of this sequence in the tagged strain compared to the untagged strain in air, compared to only 1.12 fold in 5.5% CO_2_ (Figure 4C). These results show a significant association of Rca1p to the promoter of *NCE103* in air compared to the high CO_2_ environment.

With respect to the other Rca1p associated genes, forty four of the 85 hits were specific to ambient air samples, 19 to enriched CO_2_ and 22 shared between the two conditions (Figure 4A). Rca1p binding peaks were directly associated with 4 other putative transcription factor encoding genes (*CTA24*, *TFB3*, *ZCF4* and *ZCF22*) and 2 genes involved in cell wall biosynthesis (*CHT2* encoding a chitinase and *OCH1* coding for a α-1,6-mannosyltransferase*)*. Since both *CHT2* and *OCH1* are involved in *C. albicans* virulence we selected them to examine the predicted role of Rca1p on their expression by qRT-PCR (Figure 4D) [26], [27]. Transcript levels of *CHT2* and *OCH1* were significantly higher in *rca1*Δ in air when compared to the control strain (Figure 4D). These data show that in addition to Rca1p's function as activator of CA expression in low CO_2_, this regulator can also operate as a repressor.

Remarkably, 46 of the 85 (54%) Rca1p associated genes are presently uncharacterized (Table S1 and S2). Although this observation precludes assigning a significant enrichment of genes to any cellular function, process, component (GO Term Finder, http://www.candidagenome.org/cgi-bin/GO/goTermMapper) or protein families (pfam), it suggests a broader involvement of Rca1p in CO_2_ sensing. A similar conclusion can be made following database searches with the TGACGTCA sequence involved in Cst6p binding which was retrieved in 49 promoters of *S. cerevisiae* genes. Analysis of both lists with GO Slim Mapper coupled to a chi-square test revealed a significant under-representation of genes in the process of RNA metabolic process (P-value: 0.0066) in *C. albicans* as well as in the response to chemical stimulus process for both *C. albicans* and *S. cerevisiae* (respectively P-value 0.0436 and 0.0322) while the latter was over-represented in the budding yeast contrary to *C. albicans*. However, it is important to note that the number of genes involved was relatively low (respectively 2, 4 and 6). Altogether, these results show that, except for *NCE103*, no apparent commonality of putative Rca1p targets or pathways can be identified and the large number of uncharacterized genes in the two lists of genes poses limitations to the full elucidation of the impact of these transcriptional factors on yeast cell biology. At the same time, these data could point to the existence of yet undiscovered pathways and underline the intrinsic differences between the two fungal organisms. In summary, our data establish Rca1p as the first regulator of a fungal CA and imply a wider role of this transcription factor in a new fungal CO_2_ sensing pathway.

### Rca1p regulation and role in growth, filamentation and cell wall biogenesis

**Figure 5:**
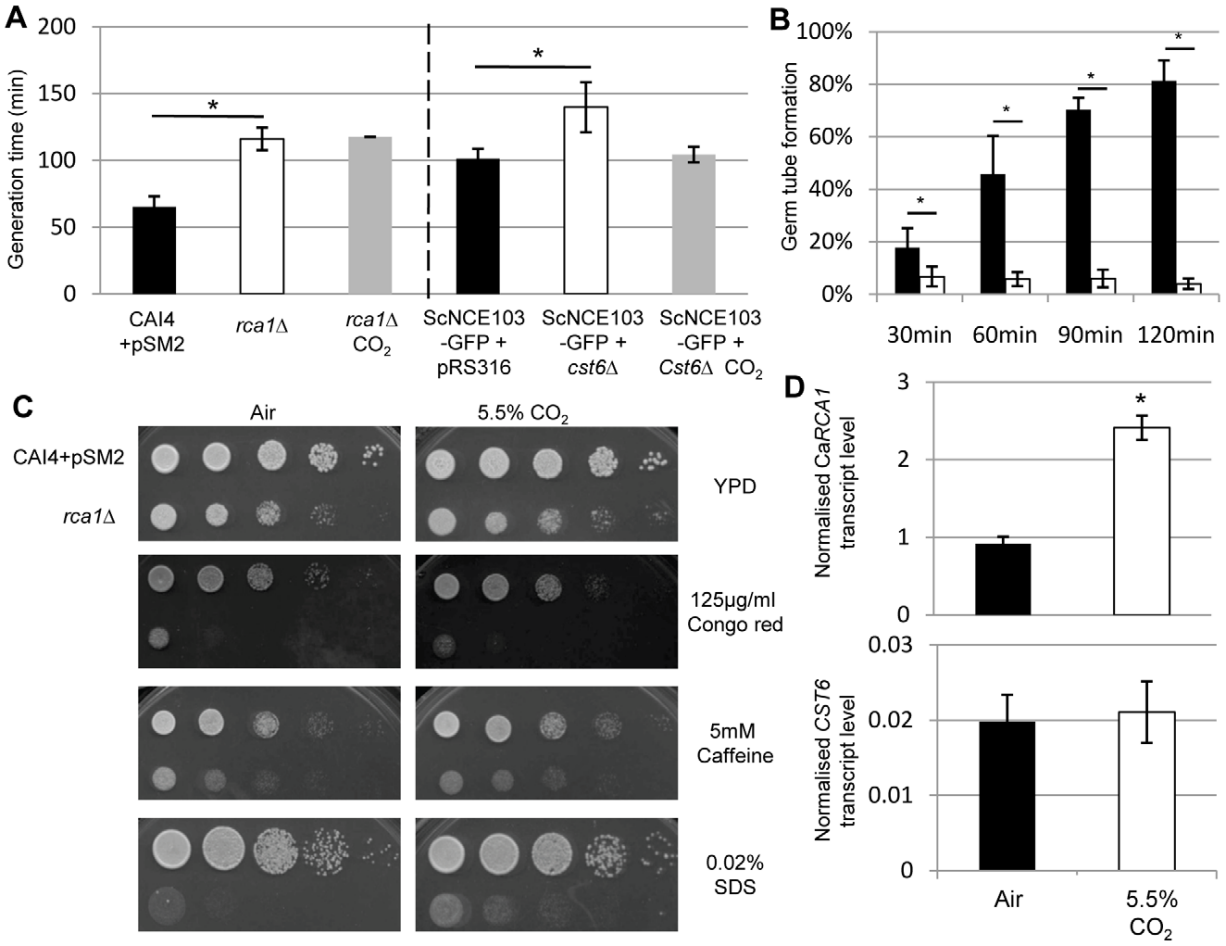
*RCA1* is involved in growth, cell wall structure, filamentation and is regulated by CO_2_. **A**) Generation time in YPD of *C. albicans* (left panel) and *S*. *cerevisiae* (right panel) control strain (black columns) and *RCA1* ortholog mutants (white columns) grown in air or 5.5% CO_2_ (grey columns). **B)** Germ tube formation in response to 5% serum of *C. albicans* control strain (black columns) and the *rca1*Δ (white columns) grown in air. **C**) Sensitivity assay of *C. albicans* control strain and *rca1*Δ. **D**) qRT-PCR using specific primers for Ca*RCA1* and *CST6* on RNA extracted from *C. albicans* (top) and *S. cerevisiae* (bottom) control strains, CAI4+pSM2 and BY4741, grown in air (black columns) or air enriched with 5.5% CO_2_ (white columns). Data are represented as mean +/− SD. Asterisk indicates statistical significance determined by two-sample *t* test (*P*≤0.05).

Since CA is critical for yeast growth in air (Figure 1), and its induction depends on Rca1p, it can be predicted that inactivation of *RCA1* should also result in a growth deficiency. Indeed we observed that *rca1*Δ has a 77% increase of its generation time compared to the control strain (Figure 5A). This phenotype is not restored in high CO_2_ pointing to a wider role of Rca1p in cell growth which could be set downstream of the CA. The enhanced growth rate of *rca1*Δ compared to *nce103*Δ is explained by residual expression of the highly effective carbonic anhydrase. We also confirmed that inactivation of *RCA1* does not lead to significant morphological alterations (Figure S6).

Our ChIP-chip data suggest a connection of Rca1p to filamentous growth and cell wall biogenesis, an observation that we confirmed by showing a strong decrease in the morphological response of *rca1*Δ to serum (Figure 5B and S6) and an increased sensitivity of *rca1*Δ to Congo red, caffeine and SDS (Figure 5C). These results set Rca1p as an important player of *C. albicans* key biological functions.

In *S. cerevisiae*, we were not able to reach identical conclusions as inactivation of *CST6* did not result in enhanced sensitivity to cell-wall perturbing agents. Additionally, only a 20% increase in generation time was observed for the *cst6*Δ mutant. Notably this phenotype was complemented by growing the strain in elevated CO_2_ (Figure 5A). These data confirm that the orthologs of *RCA1* are involved in the regulation of different cell functions further underlining their intrinsic difference emerging from the ChIP-chip and bioinformatic analysis.

Interestingly expression levels of the Rca1p orthologues is also variable between the two species (Figure 5D). Using specific primers for each species (*C. albicans* and *S. cerevisiae*), we investigated the level of *RCA1* and *CST6* transcript in low and high CO_2_ environment. In *C. albicans*, *RCA1* expression is 2.5 fold higher in hypercapnia compared to normal atmosphere (Figure 5D). In contrast, the *CST6* transcript in *S. cerevisiae* did not display any significant variation of the expression between the two conditions (Figure 5D). While the function as a regulator of carbonic anhydrase is shared among Rca1p orthologs, their regulation in response to environmental CO_2_ differs.

### Serine 124 is involved in the regulatory function of Rca1p

**Figure 6:**
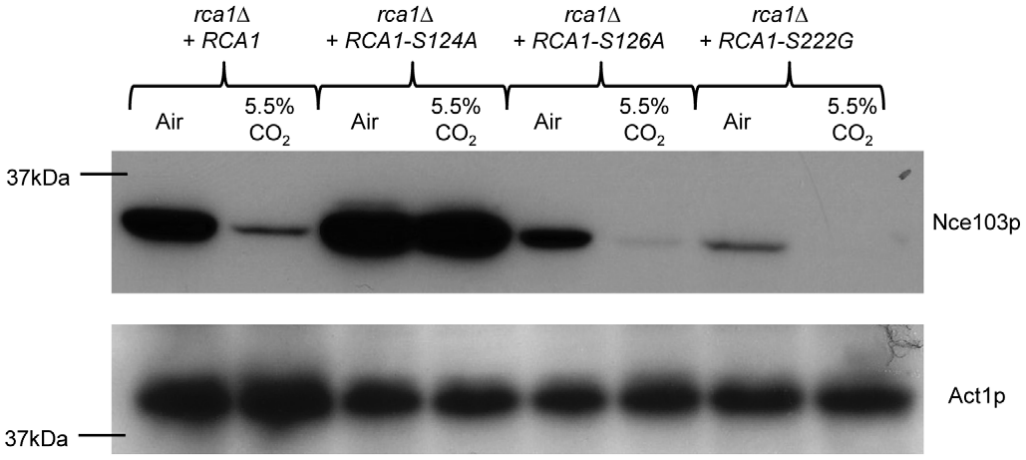
Serine 124 is involved in Rca1p function as an inducer of *NCE103*. Western blot with *C. albicans rca1*Δ complemented with a wild-type allele, an *RCA1* allele mutated in serine 124, serine 126 or serine 222.

Sequence comparison of the Rca1p orthologs from *C. albicans* and *S. cerevisiae* identified three putative sites of phosphorylation (Figure S4). We investigated the role of these residues in the function of *C. albicans* Rca1p by complementation of *rca1*Δ with constructs expressing Rca1p with a replacement of serine to alanine in position 124 and 126 (*rca1*Δ+*RCA1*−*S124A* and *rca1*Δ+*RCA1*−*S126A* respectively) or serine to glycine in position 222 (*rca1*Δ+*RCA1*−*S222G*). Loss of serine in position 126 or 222 only partially impact on the CO_2_ regulation of Nce103p expression; however mutating serine 124 lead to a striking unresponsiveness to ambient CO_2_ resulting in enhanced expression of Nce103p in both air and air enriched with 5.5% CO_2_ (Figure 6). Our results point to a critical role of serine 124 for Rca1p activity in response to CO_2_ concentrations.

### Carbonic anhydrase is differentially expressed in yeast populations

**Figure 7:**
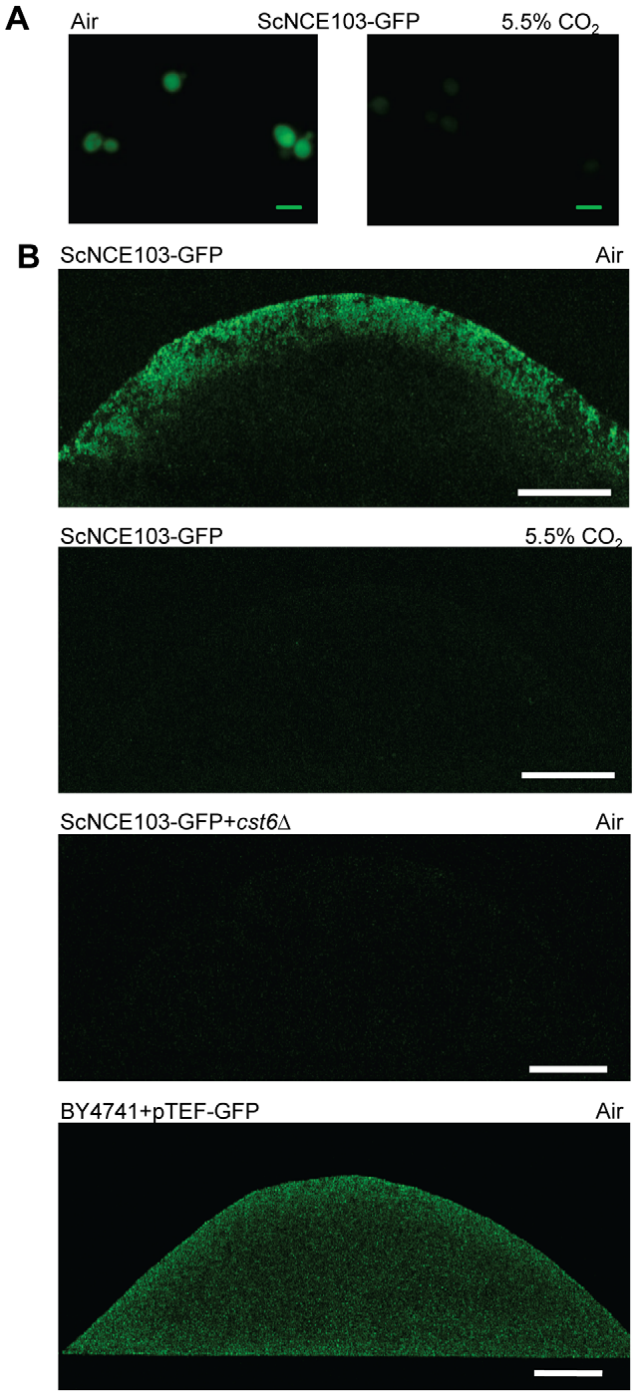
Carbonic anhydrase is differentially expressed in *S. cerevisiae* populations. **A**) Pictures of *S. cerevisiae Sc*NCE103-GFP cells grown in YPD for 4h in air (right panel) or air supplemented with 5% CO_2_ (left panel). Picture magnification x60, bar corresponds to 20 µm. **B**) Cross section of a ScNCE103-GFP colony grown in ambient air (first panel) or in air enriched with 5.5% CO_2_ (second panel), ScNCE103-GFP+*cst6Δ* (third panel) BY4741+pTEF-GFP (fourth panel) were grown in air. Bar corresponds to 100 µm.

We have previously shown that in *C. albicans* colonies metabolically-generated CO_2_ accumulates and is subsequently used to activate the adenylyl cyclase Cyr1p promoting the switch from yeast to filamentous growth essential for pathology [13]. We now substantially expand these results to entire populations of *S. cerevisiae* taking advantage of the regulation of expression of ScNce103-GFPp by CO_2_. Matching CA protein expression detected by Western blots (Figure 2A), a strong fluorescent signal was recorded in ScNCE103-GFP cells grown in ambient air but absent in air enriched with 5.5% CO_2_ (Figure 7A). Next we visualized Nce103p expression not only in individual cells but an entire fungal colony, monitoring for the first time the flux of CO_2_ in a fungal population. Using high resolution two-photon excitation confocal microscopy [28] we examined a cross-section of a ScNCE103-GFP colony grown for 4 days on solid nutrient agar. We observed that cells in the superficial layers, exposed to the low CO_2_ concentrations found in ambient air, strongly express the Nce103-GFPp construct; while the internal layers of the colony do not show any significant fluorescence (Figure 7B). Strikingly when grown in a 5.5% CO_2_ atmosphere, this gradient was absent, and no fluorescence was observed at any position in the colony. Similarly, no fluorescence was seen in *cst6*Δ, regardless of the CO_2_ concentration or the position in the colony (Figure 7B), indicating that the abscence of GFP expression in the center of the ScNCE103-GFP colony grown in air was unlikely due to a lack of viability or metabolic activity of the corresponding cells. By contrast, our positive control constitutively expressing GFP displays homogenous fluorescence through the cross-section (Figure 7B). In conclusion, our data visualizing the flux of CO_2_ inside yeast populations are in full agreement with those generated by Western blot or qRT-PCR in single cells (Figure 1C, D). Furthermore, they illustrate, with a high level of detail, the capacity of yeast to generate CO_2_ enriched micro-environments and adjust metabolic expression in a population.

## Discussion

**Figure 8:**
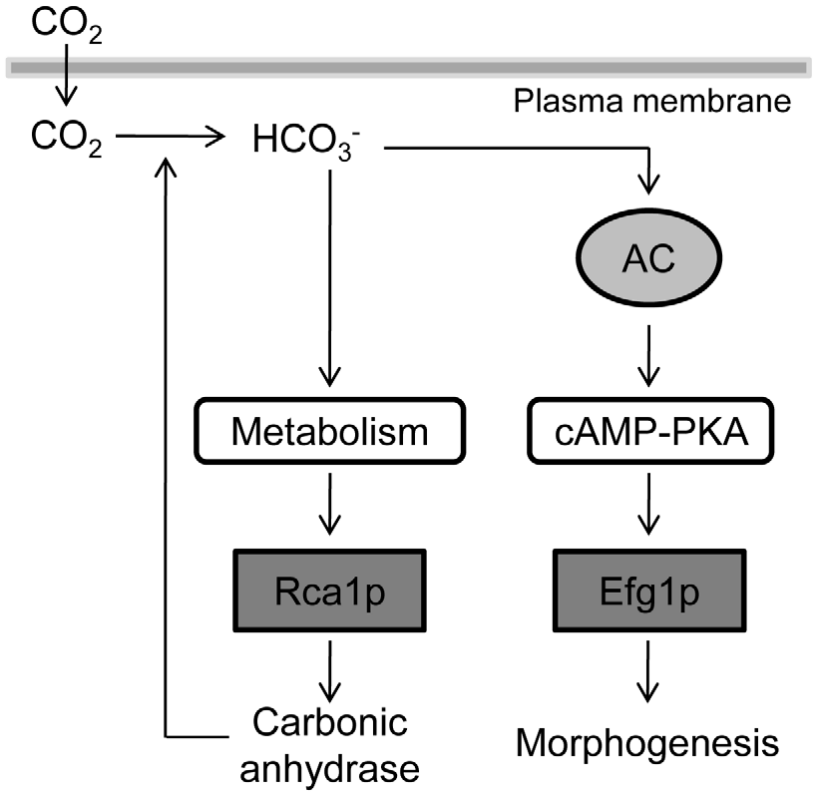
Model of CO_2_ sensing pathways in *C. albicans*. Hydration of CO_2_ into HCO_3_^−^ inside the cytoplasm occurs naturally and via the carbonic anhydrase activity. HCO_3_^−^ can enter two pathways: 1) regulating morphogenesis via the activation of the adenylyl cyclase (AC) which results in an increase of cAMP production and activation of the cAMP-PKA pathway 2) integrating metabolic demand which could signal to Rca1p the cells requirement for HCO_3_^−^ production resulting in the regulation of carbonic anhydrase expression controlling HCO_3_^−^ synthesis rate.

Carbon dioxide is a major signal in all organisms ranging from humans to fungi [7], [29]. CO_2_ regulates numerous phenotypes including virulence in the fungal pathogens of humans *C. albicans* or *C. neoformans* [7], [12]. Here we demonstrate for the first time a novel, cAMP independent, CO_2_ sensing pathway in the yeast species *C. albicans* and *S. cerevisiae*. We report that at the core of this new sensing pathway lies a bZIP transcription factor, Rca1p in *C. albicans* and its ortholog Cst6p in *S. cerevisiae*. We show that Rca1p and its orthologs regulate the expression of a major enzyme involved in fungal metabolism, CA, in response to changes in ambient CO_2_ level. CAs catalyze the synthesis of HCO_3_^−^, an essential substrate for the cell's carboxylation reaction that sustains gluconeogenesis, ureagenesis or lipogenesis [30], [31]. We hypothesize that CA is critically involved in cellular metabolism and a feedback loop involving Rca1p could exist to regulate its expression (Figure 8). Furthermore, as CA controls the level of HCO_3_^−^, the regulation of CA expression driven by cellular metabolism could have an indirect impact on the capacity of the cells to differentiate through activation of the cAMP-PKA pathway.

While HCO_3_^−^ is an essential cofactor for cellular metabolism in all fungi tested, the fungal requirement for CA is conditional, depending on the environmental CO_2_ concentration. CA mutants will not grow in ambient air where CO_2_ is scarce but will thrive in niches where the atmosphere is enriched with this gas [6], [7], [8], [9], [10]; the higher concentration allows sufficient spontaneous hydration to HCO_3_^−^ to serve as substrate for the above carboxylation reactions. Regulation of CA expression by CO_2_ has been reported for *S. cerevisiae*, *S. macrospora*, *A. fumigatus* and *A. nidulans* [8], [9], [17], [18] and we extend these observations to *C. albicans*.

Though the regulation of CA by CO_2_ has been observed in many fungi, the current work is the first to report the identification of a fungal-specific CO_2_-responsive transcription factor, Rca1p in *C. albicans* and of one of its orthologs in *S. cerevisiae*. We identified Rca1p, a previously uncharacterized bZIP-family DNA binding protein, via a functional genetic screen of a transcription factor knockout library. Rca1p functions by inducing CA protein and transcript when *C. albicans* faces low ambient CO_2_ level. Loss of CA induction in high CO_2_ level could result from a phosphorylation or another posttranslational modification on serine 124 which leads to the inability of Rca1p to bind *NCE103* promoter, integrating Rca1p in a signal transduction pathway. The impact of Rca1p in cell growth and cell wall biogenesis, independently of CO_2_ concentration, points to a general involvement of Rca1p in the cellular metabolism of *C. albicans*. Rca1p function as CA regulator is conserved in *S. cerevisiae*, though they also have additional functions: Cst6p has already been shown to be involved in functions such as growth on non-optimal carbon sources [23], and our results now highlight the importance of Cst6p in cellular metabolism via its role as an inducer of CA. However, the impact of Cst6p on cell physiology differs when compared to *C. albicans* since the *cts6*Δ mutant growth defect in air is complemented by addition of environmental CO_2_ for *S. cerevisiae*. Furthermore the observation that *RCA1* expression itself is regulated by CO_2_ underlines the importance of this regulator outside the scope of CA expression. Notably, Rca1p orthologs can be identified in *S. macrospora*, *A. fumigatus* and *A. nidulans* known to posses CA's which expression is influenced by ambient CO_2_ level [8], [9].

Importantly, Rca1p is distinct at both the sequence and functional level from the best characterized regulator of eukaryotic CAs, HIF-1α, which induces human CA IX expression in response to hypoxia [15], [32]. CA IX leads to extracellular acidification of hypoxic tissue, and is as such abundant in tumors [33]. By contrast *C. albicans NCE103*, is not regulated by either hypoxia or changes in pH ([34]; Figure S7).

Similar to HIF-1α, which binds to the HRE motif present in CA IX promoter [32], the regulation of *NCE103* by Rca1p appears to be direct. ChIP-chip and ChIP-qPCR confirmed that Rca1p binds to the CA promoter of *C. albicans,* specifically under low ambient CO_2_, leading to the induction of *NCE103* expression. An additional 84 genes were associated with Rca1p, suggesting a much broader role of this new CO_2_ signaling pathway. Although the majority of these genes are currently of unknown function, in depth analysis of two (*OCH1* and *CHT2*) showed Rca1p's potential to act as both an inducer and repressor of gene expression. This dual function could also be explained by an ability of Rca1p to recruit different co-factors at the associated loci. Using SCOPE [35] and other predictive programs, we obtained a relatively low number of results with significant value regarding binding sites or processes associated to Rca1p and Cst6p. These observations could be due to a higher complexity in the binding motif of Rca1p to the DNA, as well as on our limited knowledge about genes function (75% of ORF in *C. albicans* are still considered as uncharacterized; http://www.candidagenome.org). Reflecting the 235 million years [36] of separation between the *Candida* and *Saccharomyces* clade, we observed profound divergence in the associated genes of Rca1p and Cst6p. However, the CA remains a conserved target for both species. Such a fundamental wiring re-arrangement between closely related transcriptional factor of *C. albicans* and *S. cerevisiae* has already been reported [37].

In *S. cerevisiae*, CO_2_ regulation of CA expression by Cst6p involves the TGACGTCA palindrome motif. Database searches with this sequence retrieved the motif in 49 promoter regions of *S. cerevisiae*, and 40 promoters of *C. albicans*. While this motif is absent in the *NCE103* promoter in *C. albicans*, it is present in a single gene associated to Rca1p (*orf19.4246*) demonstrating the lack of motifs conservation between the two species. Interestingly, the promoter of human CA IX presents a bZIP binding motif, TGAGTCA [38], which is closely related to the one identified in *S. cerevisiae*. Furthermore this motif is the binding sequence of the oncogen C-jun in human, which presents some sequence similarity with Rca1p (Score 42.7, e value 0.003), particularly around the bZIP domain. To date, the expression of CA IX in response to CO_2_ changes in the body has not been investigated. Our results may point to an additional level of regulation in human CA's; in fact HIF-1α sole predominance in CA IX regulation has recently been questioned [39].

The regulation of CA by CO_2_ is likely to be complex; however, using a new antibody that we generated, we show that CO_2_ affects CA proteins levels dramatically – highly induced in normal and undetectable when cells are grown in an elevated CO_2_ atmosphere. However, CA transcript levels were only decreased by 50% relative to ambient air. This type of regulation may suggest additional levels of post-transcriptional control on CA messenger. Maintaining CA transcripts in high CO_2_ level would allow a shortened response in CA enzyme synthesis when cells encounter a switch from high to low CO_2_ atmosphere, thus ensuring sufficient supply of the essential HCO_3_^−^ ion.

We have begun to uncover the complex physiology associated with variations in CO_2_ concentrations. *C. albicans* cells phagocytosed by macrophages induce CA, as seen by qRT-PCR, despite being in a high CO_2_ (5%) environment, and this is Rca1p-dependent. This suggests that the phagocyte might restrict CO_2_ availability, as it does for other nutrients. Furthermore, using high resolution two-photon excitation confocal microscopy with GFP-tagged CA's we visualize for the first time the impact of CO_2_ build-up on gene expression in single cells but also in entire fungal populations. The data presented in this report not only confirm previous observations made in *C. albicans* that CO_2_ is compartmentalized in yeast populations, specifically inducing developmental change [13], but importantly connects micro-environments enriched in CO_2_ to metabolic specialization of individual members of a fungal populations. However, it is important to also consider the possibility that an unknown, CO_2_-independent, pathway is involved in the regulation of Nce103p at colony level. CA regulation is exquisitely sensitive to change in CO_2_ availability in both *S. cerevisiae* and *C. albicans* (Figure S8) and our results may be highly applicable to a range of conditions in which fungi expand and act as populations rather than individual cells including the formation of drug-resistant biofilms in pathogenic yeasts such as *C. albicans*. For *S. cerevisiae*, biofilms are naturally isolated on fruit surface (grape), and have a major application in industrial fermentation [40], [41].

The effects of CO_2_ on fungal physiology are integrated through more than one regulatory circuit. We previously showed that elevated CO_2_/HCO_3_^−^ is sensed by the adenylyl cyclase via a lysine residue of the enzymes catalytic core, increasing the production of the second messenger cAMP, thus linking adenylyl cyclase and CA in fungal CO_2_ sensing [7], [12]. Adenylyl cyclase/cAMP are particularly important mediators of fungal virulence determinants. Although there is cross-talk between the activities of the two enzymes, we now show that the CO_2_ control of CA expression acts independently from the cAMP-PKA pathway. This mechanism has to be compared to the identification of a cAMP-independent CO_2_-sensitive pathway involved in white opaque switching which results in Wor1p phosphorylation [42]. However, the putative overlap of these two uncharacterized pathways remains to be defined.

In conclusion, carbon dioxide is sensed in yeast by two independent pathways. One, previously described by us, involves the fungal adenylyl cyclase and cAMP [12]. We have now identified a second pathway and found the transcriptional regulator, Rca1p, at the core of the new pathway in yeast. Activation or inactivation of the transcription factor may involve phosphorylation which ultimately programs cellular metabolism to allow optimal adaptation to the environment inside a macrophage in the case of a fungal pathogen or yeast populations. Investigating the function of the additional Rca1p-associated genes will bring better understanding on how organisms sense the universal gas carbon dioxide.

## Materials and Methods

### Strains

All strains and plasmids used or constructed in this study are reported in supporting information (Table S3, S4, S5), as is the composition of the respective media and additional protocols (Protocol S1). *C. albicans* were incubated at 37°C, or 30°C for *S. cerevisiae*, either in ambient air or air enriched with 5.5% (vol/vol) atmospheric CO_2_ (Infors HT Minitron) when required.

### Protein extraction and Western blot

Strains were inoculated in 50mL of YPD at OD_600_ 0.1 and grown at the suitable temperature in air or air enriched with CO_2_. After 4h, cells were collected and quickly frozen. Samples were disrupted using a Mikro-dismembrator S (Sartorius) and resuspended in 500 µl lysis buffer (50 mM HEPES, 150 mM NaCl, 5 mM EDTA, 1% Triton X-100, protease inhibitor [Roche]). Protein concentrations were quantified using Bradford reagent (Sigma). 30 µg of protein were loaded for each sample on a 12% SDS-acrylamide/bis-acrylamide gel, and proteins were transferred to a PVDF membrane (Millipore). Membranes were incubated with the appropriate antibodies diluted as follows: anti-NCE103 at 1∶500, anti-LacZ at 1∶1000 (Millipore), anti-GFP at 1∶2500 (Roche) or anti-Act1p 1∶1000 (Sigma). This was followed by a second incubation with a peroxidase tagged antibodies of goat anti-rabbit, diluted 1∶2000, (Sigma) for the anti-NCE103, anti-LacZ and anti-Act1 primary antibodies, while a goat anti-mouse, diluted 1∶5000 (Sigma), was used for the anti-GFP primary antibody. Luminol Electrochemiluminescence was used to detect signal on the membrane.

### qRT-PCR

Culture and samples were prepared in an identical manner as for the protein extraction, apart from total RNA extraction which was carried out with the RNeasy Kit (Qiagen) according to the manufacturer's recommendations. Transcripts level were determined by semi quantitative RT-PCR using the iScript One-Step RT-PCR Kit with Syber Green (BioRad). Levels were normalised to *ACT1* from the respective species, and calculated using the Delta C(t) analysis of the Opticon Monitor 3 software (Bio-Rad). Values are represented as mean +/− SD from three independent experiments.

### Chromatin Immuno Precipitation on Chip

50ml cultures in YPD of strains *RCA1*/*rca1*Δ+*RCA1* (untagged) and *RCA1*/*rca1*Δ+*RCA1*−*HA_3_* (tagged) were inoculated at OD_600_ 0.1 by overnight culture and incubated 4h at 37°C in air or air enriched with 5.5% CO_2_, 140 rpm. Three independents cultures were grown for each strain in both conditions. The subsequent steps of DNA cross-linking, DNA shearing, chromatin immunoprecipitation (ChIP), DNA labeling with Cy3 or Cy5 dyes, hybridization to intergenic DNA microarrays, and data analysis were conducted exactly as described [42]. Cy5-labeled DNA from the tagged strain (*RCA1*/*rca1*Δ+*RCA1*−*HA_3_*) and the corresponding Cy3-labeled DNA from the untagged control strain (*RCA1*/*rca1*Δ+*RCA1*) were mixed and hybridized to a *C. albicans* whole-genome tiled oligonucleotide DNA microarray [24]. After hybridization and scanning of the slides (*n* = 3 for each condition), results were process [43]. Quantile normalization was applied to the data [25]. The parameters used were: a window size of 400 bp, a maximum genomic distance of 60 bp, and a minimum length of 120 bp. The replicate data were combined, and peak finding (i.e., determining the Rca1-HA_3_p binding sites) was done using a pseudomedian signal threshold of at least 1.5 fold and a *P* value cutoff of 0.01 [25], [44].

### ChIP-qPCR

Chromatin Immuno Precipitations were processed according to the above protocol with the identical strains *RCA1*/*rca1*Δ+*RCA1* (untagged) and *RCA1*/*rca1*Δ+*RCA1*−*HA_3_* (tagged) and growth conditions. The resulting purified DNA was used in quantitative PCR using SYBR Green Master Mix (Applied Biosystems, Inc.) with primers: Ca-ChIP-NCE103-F/Ca-ChIP-NCE103-R for the detection of the *NCE103* promoter (a 195bp region identified to be significantly associated with Rca1p) and Ca-ChIP-ACT1-F/Ca-ChIP-ACT1-R for the control *ACT1* promoter, a gene without known association for Rca1p. Levels of detection were normalized to *ACT1* and calculated using the Delta Delta C(t) method. Values are represented as mean +/− SD from two independent experiments.

### Macrophage co-culture experiments

*C. albicans* wild-type (SC5314) and *rca1Δ* were grown to log-phase in YPD medium, washed in water, and counted. They were then incubated with J774A.1 macrophages at an MOI of 2∶1 (*C. albicans*: macrophages) in RPMI+10% FBS at 37°C in 5% CO_2_ in 750 cm^3^ vented flasks. Control cells were grown in the same media without macrophages at 37°C in 5% CO_2_. After incubation for one hour, the flasks were rinsed with PBS, then cells were collected by scraping into ice cold water and transferred to conical tubes. They were washed twice more with water, then pellets were frozen on dry ice. RNA was prepared using the Turbo DNA-free kit (Ambion). 50 ng of total RNA were used for each qRT-PCR reaction using the Power SYBR Green reaction system (Invitrogen). Actin (*ACT1*) was used as the normalization control. Primers are listed in supplemental data.

### Microscopy

ScNCE103-GFP cells were observed with a Olympus IX-81 fluorescence microscope with a 150 W xenon-mercury lamp and an Olympus 60X Plan NeoFluor oil-immersion objective.

For high resolution two-photon excitation confocal microscopy of entire yeast colonies of ScNCE103-GFP, ScNCE103−GFP+*cst6*Δ and BY4741+pTEF−GFP, cells were grown for 4 days on YPD at 28°C. Colonies were then embedded in low-gelling agarose (Sigma-Aldrich) directly on the plates [28]. After solidification, agarose-embedded colonies (an area of approximately 10×10 mm) were sectioned vertically down the middle and transferred to the cover glass. All samples on the cover slip were enclosed with a thick agarose layer to prevent them from drying. Image acquisition was realized following published protocol [28], using 20 x/0.7 water immersion planachromat objective.

### Statistical analysis

Statistical analyses were performed using Student's t test. P values are indicated as detailed in the figure legends. Error bars in figures represent SD.

## Supporting Information

Figure S1 **Carbonic anhydrase expression is independent of the cAMP-PKA pathway.** Using our anti-Nce103p and anti-GFP antibodies, carbonic anhydrase signals are shown in western blots from *C. albicans* (top) and *S. cerevisiae* (bottom). Proteins were extracted from cells grown in YPD for 4h in air (with or without addition of 10 mM dbcAMP to the culture medium) or air enriched with 5.5% CO_2_. Yeast carbonic anhydrase expression is not influenced by the addition of dbcAMP. The same samples were probed with an anti-actin antibody as control.
(TIF)

Figure S2 **Strains verification in** ***C. albicans*** **and** ***S. cerevisiae*** **. A**) Southern blot where genomic DNA from strain CAI4+pSM2 (1), *rca1Δ*+*RCA1* (2), *rca1Δ*+*RCA1*−*HA_3_* (3), *rca1Δ* (4), *rca1Δ*/*RCA1*+*RCA1* (5 and 6) and *rca1Δ*/*RCA1*+*RCA1*−*HA_3_* (7) were digested by *Sac*I, migrated on agarose gel and transfered onto nitrocellulose membrane. Using a *RCA1* probe, expected bands were observed with a signal at 3.8kbp for the *RCA1* allele, 4.6kbp for *rca1Δ*, 8.1 and 8 kbp for the introduction of the pSM2-RCA1 and pSM2-RCA1-HA_3_ alleles respectively. **B**) Diagnostic PCR products with primers NCE103-Verif-F and ScNCE-end using genomic *S. cerevisiae* DNA of control strain BY4741 (lane 1) and Sc*nce103Δ* (lane 3) as template, or primers Nce.ko.kan-F and NCE103-Verif-R with the respective template on lane 2 and 4. First set of primers hybridize on each side of the cassette, second set confirm presence of the cassette at the right locus. **C**) Diagnostic PCR products with primers CST6-Verif-F and CST6-Verif-R using genomic *S. cerevisiae* DNA of control strain ScNCE103-GFP (lane 1) and ScNCE103-GFP+*cst6Δ* (lane 3) as template, or primers ScCST6.ko.kan-F and CST6-Verif-R with the respective template on lane 2 and 4. First set of primers hybridize on each side of the cassette, second set confirm presence of the cassette at the right locus.
(TIF)

Figure S3 **Verification of gene expression by qRT-PCR. A**) qRT-PCR using *RCA1* specific primers and RNA extracted from *C. albicans* control strain and the *RCA1* mutant grown in air. The *rca1Δ* strains show no significant level of *RCA1* transcript. **B**) qRT-PCR using Sc*NCE103* (top) and *CST6* (bottom) specific primers and RNA extracted from the *S. cerevisiae* controls, Sc*NCE103* mutant and *CST6* mutant strain grown in air enriched with 5.5% CO_2_ (top panel) or air (bottom panel). Both mutants show no significant level of Sc*NCE103* and *CST6* transcript compared to the control strain. **C**) qRT-PCR using *ORF19.6103* (top) and *MVD* (bottom) specific primers and RNA extracted from *C. albicans* control and the *RCA1* mutant grown in air (black columns) or air enriched with 5.5% CO_2_ (white columns). Expression of both genes is not significantly different between the control and mutant strain. Data are represented as mean +/− SD from three independent experiments. Asterisk indicates statistical significance determined by two-sample *t* test (*P*≤0.05).
(TIF)

Figure S4 **Protein alignment of Rca1p and Cst6p sequences.** Alignment of *C. albicans* Rca1p (C.a.) and *S. cerevisiae* Cst6p (S.c.) sequences by ClustalW2 (http://www.ebi.ac.uk/Tools/clustalw2/index.html). “*”, “:” and “.” respectively means that the residues of that column are identical in the two sequences, that conserved substitutions occurred, or that semi-conserved substitutions are observed. The bZIP motifs (bold) are present in the C-terminus of each protein. 3 conserved putative serine sites for phosphorylation (underlined) are shown (http://www.cbs.dtu.dk/services/NetPhosYeast/).
(TIF)

Figure S5 **The** ***RCA1*** **heterozygous mutant and complemented strains display a wild-type pattern of Nce103p expression.** Carbonic anhydrase signals are shown in western blots from the *C. albicans* control, *RCA1* heterozygous mutant, and the complemented strains. All strains display an identical profile of Nce103p expression. The same samples were probed with an anti-actin antibody as control.
(TIF)

Figure S6 ***RCA1*** **inactivation does not impact on cells morphology.** Control (CAI4+pSM2) and *rca1* mutant (*rca1*Δ) strain were grown for 2h at 37°C in YPD supplemented or not with 5% horse serum. Representative pictures show identical morphology for both strains in YPD and confirm the inability of the *rca1* mutant to differentiate into hyphae. Bar corresponds to 5 µm.
(TIF)

Figure S7 ***C.albicans*** **Nce103p is not regulated by environmental pH.** Western blots showing carbonic anhydrase signals from the *C. albicans* control strain grown in YPD buffered at pH 4 or pH 7 for 4h in air. In both conditions, Nce103p signals are identical.
(TIF)

Figure S8 **Nce103p induction is exquisitely sensitive to ambient CO_2_ availability.** Using appropriate antibodies, carbonic anhydrase signals are shown in western blots from *S. cerevisiae* (top) and *C. albicans* (bottom). Proteins were extracted from cells grown in YPD for 4h in air or air enriched with 0.5 or 1% CO_2_. Nce103p signals are detectable in ambient air, and air enriched with 0.5% CO_2_.
(TIF)

Protocol S1 **Detailed protocols about media used in this study, strains and plasmids construction, yeast transformations, Southern blot analysis and the generation of** ***C. albicans*** **Nce103p antibodies, as well as supporting references.**
(DOC)

Table S1 **Rca1p-HA_3_ binding in air dataset.** The following criteria were used: Log_2_ pseudo-median signal intensity threshold of ≥0.5 and p-value cut-off of ≤0.01 [12]. **Contig19#:** The Contig19 number on which a given binding peak is detected using the Tilescope software [12]. **Location:** Position of the binding peak in the corresponding Contig19 DNA sequence. **Log2 pseudo-median signal intensity:** Log_2_-transformed pseudo-median signal intensity of Rca1p-HA_3_-binding at the corresponding location. **Target:** orf19 nomenclature according to the *C. albicans* Assembly 19 of Rca1p-HA_3_ target gene, based on the location of the locus relative to the binding peak. Absence of information indicates that binding peaks are not clearly associated with promoter of ORFs. If the peak was found in the promoter region common to two adjacent ORFs, the two possible predicted target genes are shown, separated by “and”. **CGD Gene name:** Gene name of the corresponding target gene according to the *Candida* Genome Database (CGD) (www.candidagenome.org). **Description:** Gene description according to CGD.
(DOCX)

Table S2 **Rca1p-HA_3_ binding in 5.5% CO_2_ dataset.** The following criteria were used: Log_2_ pseudo-median signal intensity threshold of ≥0.5 and p-value cut-off of ≤0.01 [12]. **Contig19#:** The Contig19 number on which a given binding peak is detected using the Tilescope software [12]. **Location:** Position of the binding peak in the corresponding Contig19 DNA sequence. **Log2 pseudo-median signal intensity:** Log_2_-transformed pseudo-median signal intensity of Rca1p-HA_3_-binding at the corresponding location. **Target:** orf19 nomenclature according to the *C. albicans* Assembly 19 of Rca1p-HA_3_ target gene, based on the location of the locus relative to the binding peak. Absence of information indicates that binding peaks are not clearly associated with promoter of ORFs. If the peak was found in the promoter region common to two adjacent ORFs, the two possible predicted target genes are shown, separated by “and”. **CGD Gene name:** Gene name of the corresponding target gene according to the *Candida* Genome Database (CGD) (www.candidagenome.org). **Description:** Gene description according to CGD.
(DOCX)

Table S3 **Plasmids used and construct in this study.**
(DOCX)

Table S4 **Strains used and constructed in this study.**
(DOCX)

Table S5 **Primers used in this study.**
(DOCX)
