# Supplementary figures and images for: The bZIP Transcription Factor Rca1p Is a Central Regulator of a Novel CO2 Sensing Pathway in Yeast
Source: PLoS Pathog. 2012 Jan 12;8(1):e1002485. doi: 10.1371/journal.ppat.1002485 (PMC3257301; doi:10.1371/journal.ppat.1002485)

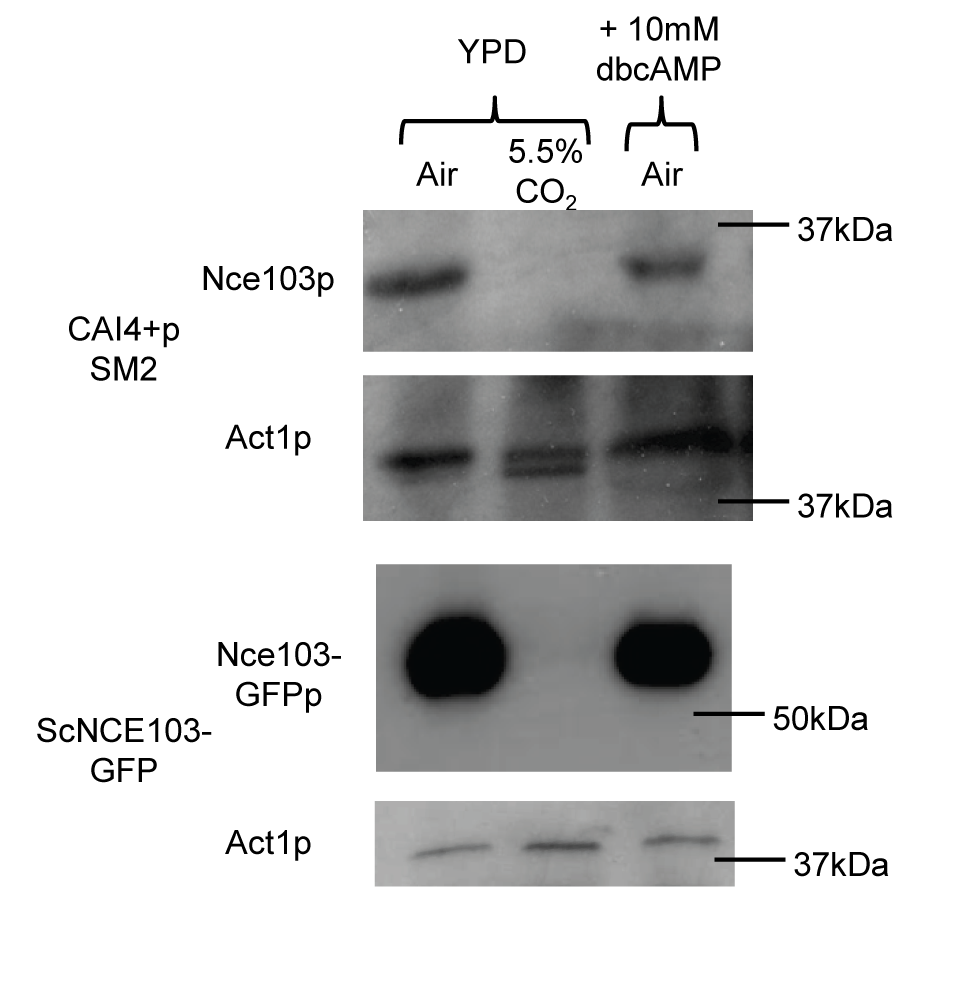

Supplement: Figure S1 — Carbonic anhydrase expression is independent of the cAMP-PKA pathway. Using our anti-Nce103p and anti-GFP antibodies, carbonic anhydrase signals are shown in western blots from C. albicans (top) and S. cerevisiae (bottom). Proteins were extracted from cells grown in YPD for 4h in air (with or without addition of 10 mM dbcAMP to the culture medium) or air enriched with 5.5% CO2. Yeast carbonic anhydrase expression is not influenced by the addition of dbcAMP. The same samples were probed with an anti-actin antibody as control. (TIF) [file ppat.1002485.s001.tif]

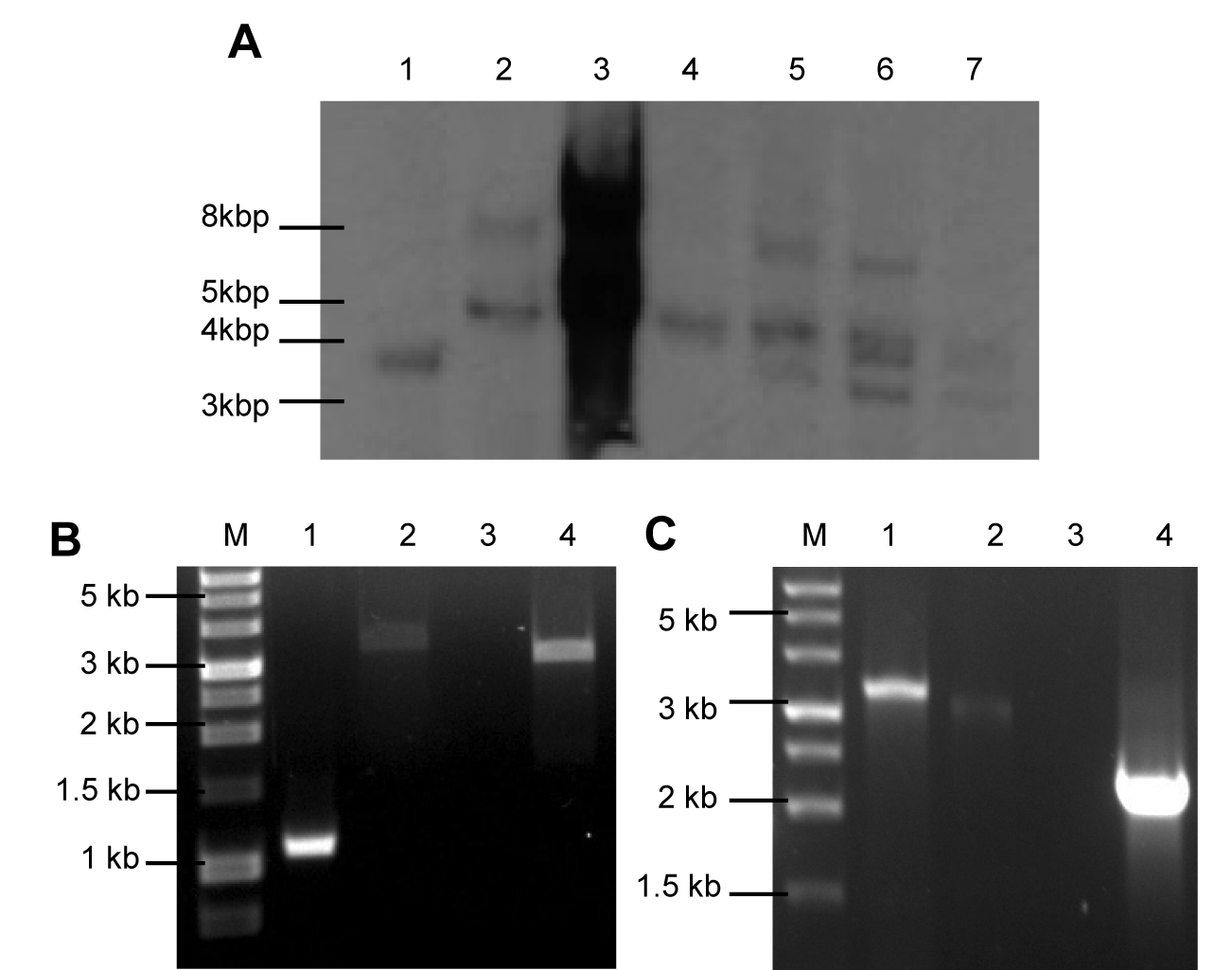

Supplement: Figure S2 — Strains verification in C. albicans and S. cerevisiae . A) Southern blot where genomic DNA from strain CAI4+pSM2 (1), rca1Δ+RCA1 (2), rca1Δ+RCA1−HA3 (3), rca1Δ (4), rca1Δ/RCA1+RCA1 (5 and 6) and rca1Δ/RCA1+RCA1−HA3 (7) were digested by SacI, migrated on agarose gel and transfered onto nitrocellulose membrane. Using a RCA1 probe, expected bands were observed with a signal at 3.8kbp for the RCA1 allele, 4.6kbp for rca1Δ, 8.1 and 8 kbp for the introduction of the pSM2-RCA1 and pSM2-RCA1-HA3 alleles respectively. B) Diagnostic PCR products with primers NCE103-Verif-F and ScNCE-end using genomic S. cerevisiae DNA of control strain BY4741 (lane 1) and Scnce103Δ (lane 3) as template, or primers Nce.ko.kan-F and NCE103-Verif-R with the respective template on lane 2 and 4. First set of primers hybridize on each side of the cassette, second set confirm presence of the cassette at the right locus. C) Diagnostic PCR products with primers CST6-Verif-F and CST6-Verif-R using genomic S. cerevisiae DNA of control strain ScNCE103-GFP (lane 1) and ScNCE103-GFP+cst6Δ (lane 3) as template, or primers ScCST6.ko.kan-F and CST6-Verif-R with the respective template on lane 2 and 4. First set of primers hybridize on each side of the cassette, second set confirm presence of the cassette at the right locus. (TIF) [file ppat.1002485.s002.tif]

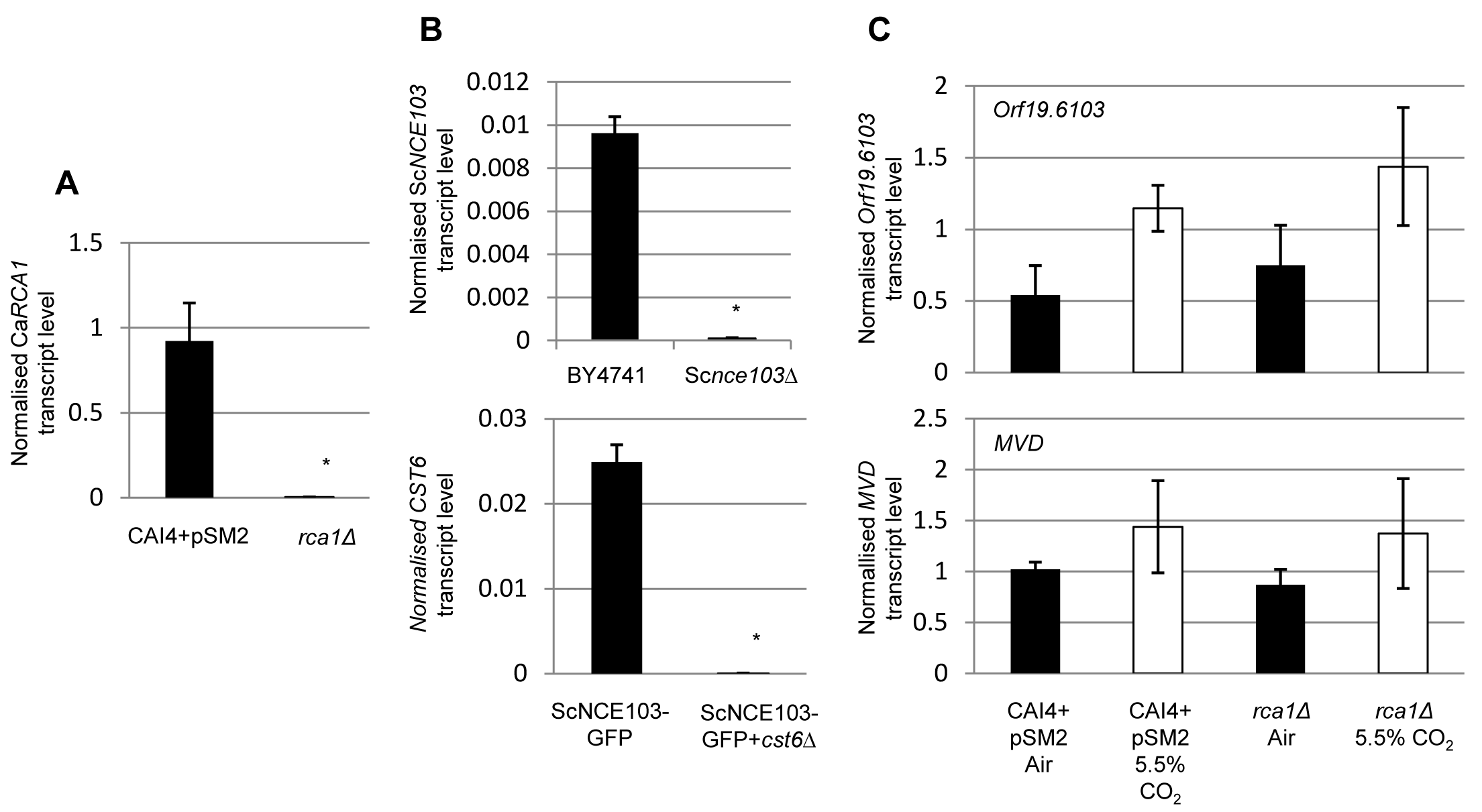

Supplement: Figure S3 — Verification of gene expression by qRT-PCR. A) qRT-PCR using RCA1 specific primers and RNA extracted from C. albicans control strain and the RCA1 mutant grown in air. The rca1Δ strains show no significant level of RCA1 transcript. B) qRT-PCR using ScNCE103 (top) and CST6 (bottom) specific primers and RNA extracted from the S. cerevisiae controls, ScNCE103 mutant and CST6 mutant strain grown in air enriched with 5.5% CO2 (top panel) or air (bottom panel). Both mutants show no significant level of ScNCE103 and CST6 transcript compared to the control strain. C) qRT-PCR using ORF19.6103 (top) and MVD (bottom) specific primers and RNA extracted from C. albicans control and the RCA1 mutant grown in air (black columns) or air enriched with 5.5% CO2 (white columns). Expression of both genes is not significantly different between the control and mutant strain. Data are represented as mean +/− SD from three independent experiments. Asterisk indicates statistical significance determined by two-sample t test (P≤0.05). (TIF) [file ppat.1002485.s003.tif]

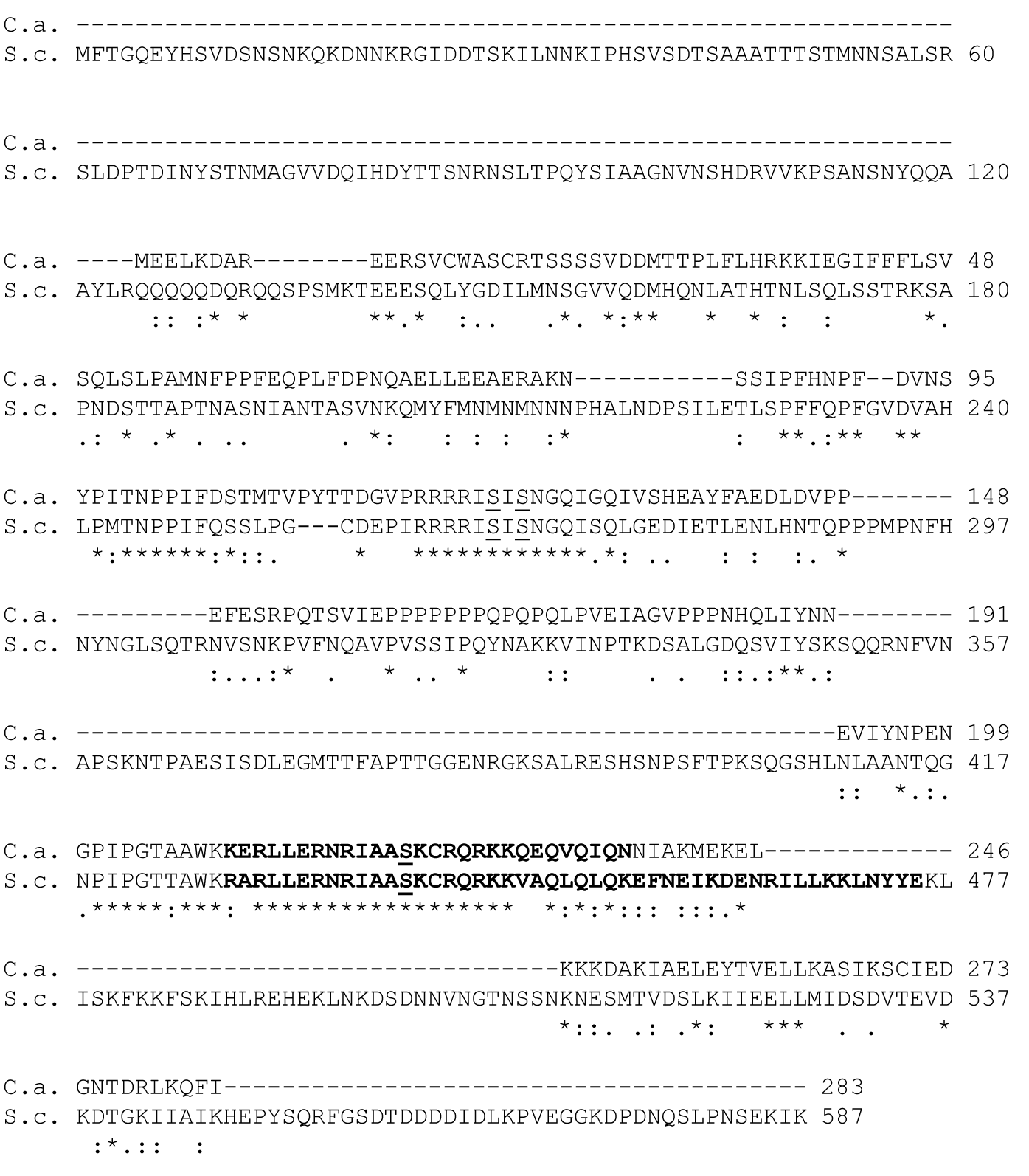

Supplement: Figure S4 — Protein alignment of Rca1p and Cst6p sequences. Alignment of C. albicans Rca1p (C.a.) and S. cerevisiae Cst6p (S.c.) sequences by ClustalW2 (http://www.ebi.ac.uk/Tools/clustalw2/index.html). “*”, “:” and “.” respectively means that the residues of that column are identical in the two sequences, that conserved substitutions occurred, or that semi-conserved substitutions are observed. The bZIP motifs (bold) are present in the C-terminus of each protein. 3 conserved putative serine sites for phosphorylation (underlined) are shown (http://www.cbs.dtu.dk/services/NetPhosYeast/). (TIF) [file ppat.1002485.s004.tif]

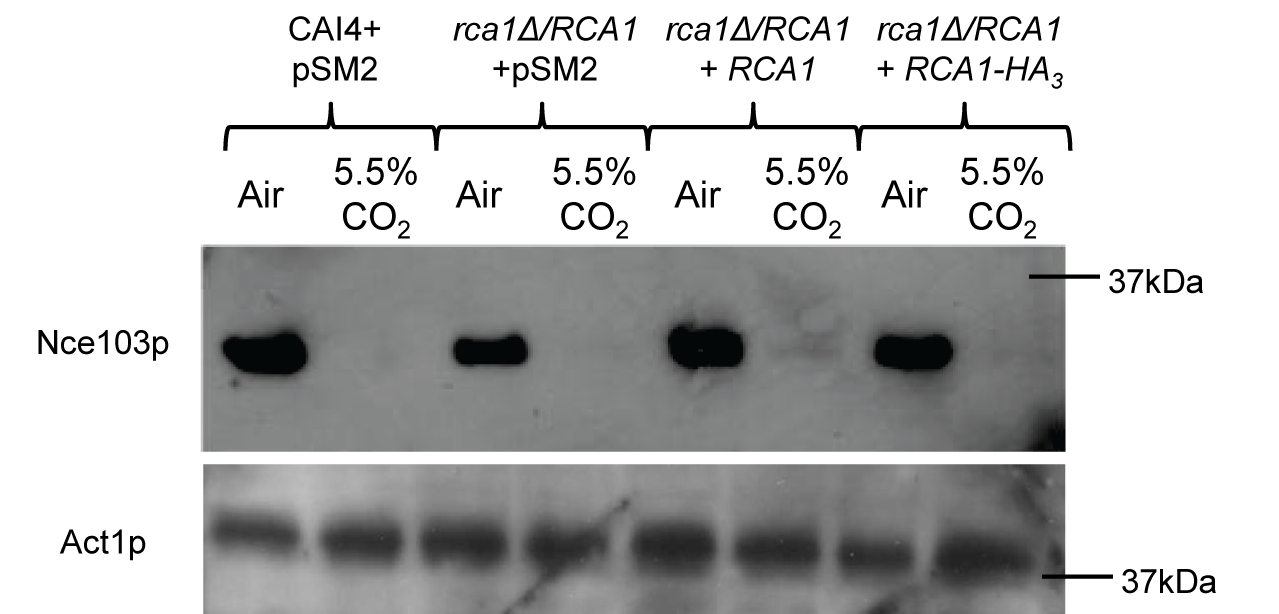

Supplement: Figure S5 — The RCA1 heterozygous mutant and complemented strains display a wild-type pattern of Nce103p expression. Carbonic anhydrase signals are shown in western blots from the C. albicans control, RCA1 heterozygous mutant, and the complemented strains. All strains display an identical profile of Nce103p expression. The same samples were probed with an anti-actin antibody as control. (TIF) [file ppat.1002485.s005.tif]

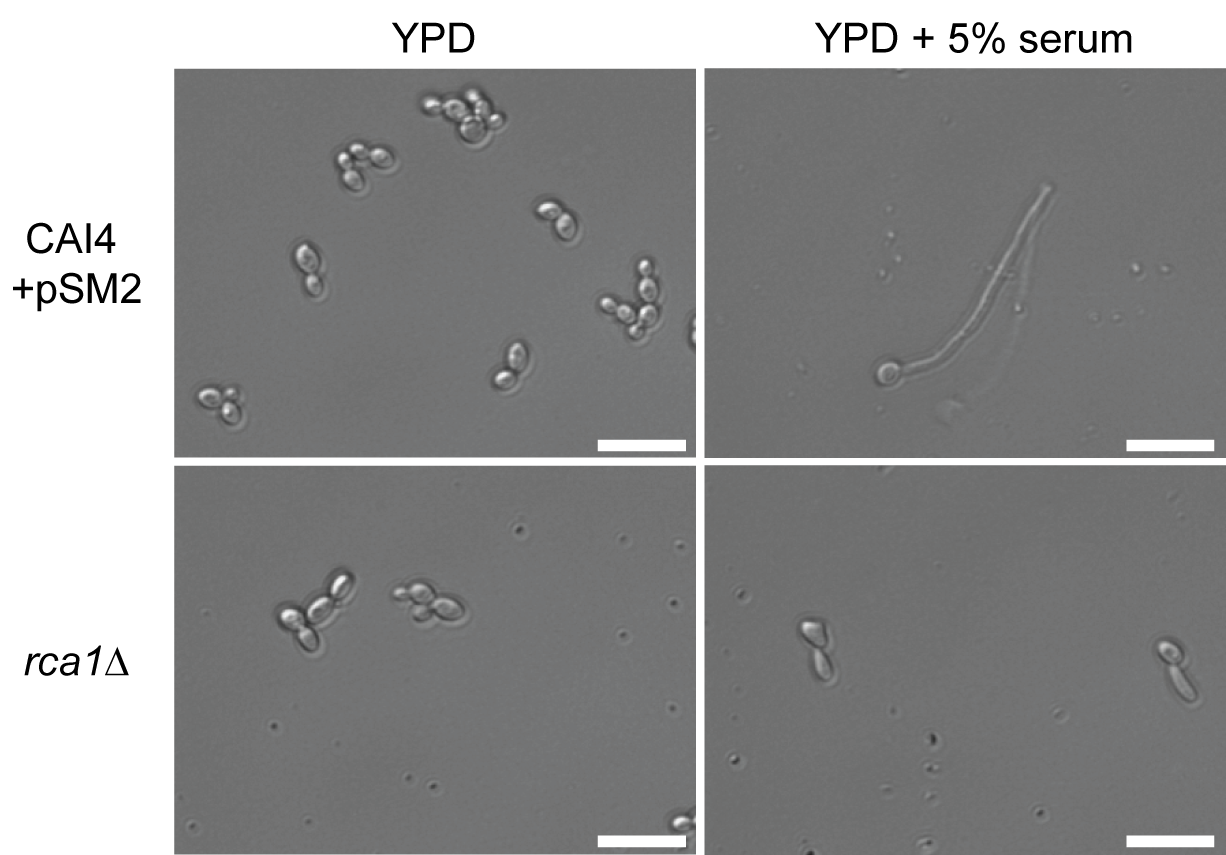

Supplement: Figure S6 — RCA1 inactivation does not impact on cells morphology. Control (CAI4+pSM2) and rca1 mutant (rca1Δ) strain were grown for 2h at 37°C in YPD supplemented or not with 5% horse serum. Representative pictures show identical morphology for both strains in YPD and confirm the inability of the rca1 mutant to differentiate into hyphae. Bar corresponds to 5 µm. (TIF) [file ppat.1002485.s006.tif]

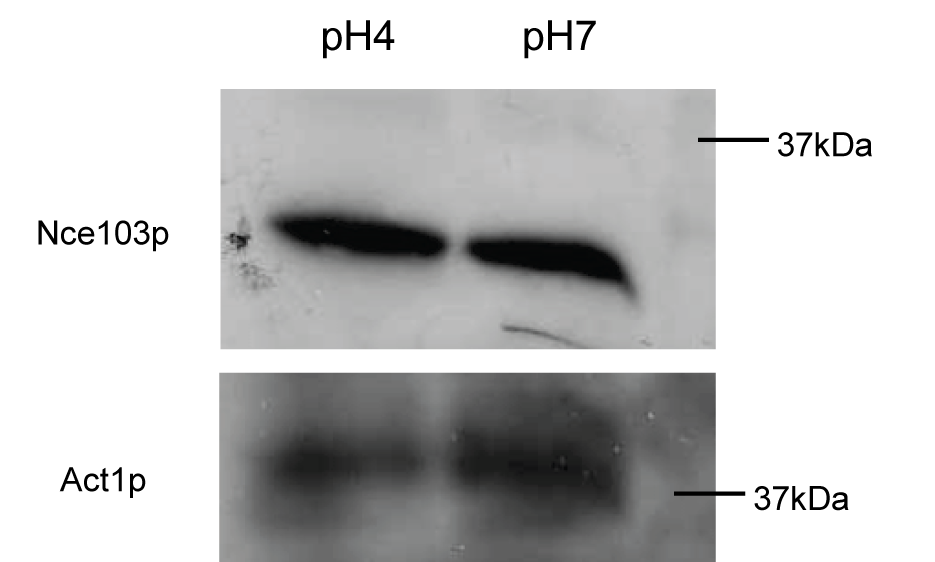

Supplement: Figure S7 — C.albicans Nce103p is not regulated by environmental pH. Western blots showing carbonic anhydrase signals from the C. albicans control strain grown in YPD buffered at pH 4 or pH 7 for 4h in air. In both conditions, Nce103p signals are identical. (TIF) [file ppat.1002485.s007.tif]

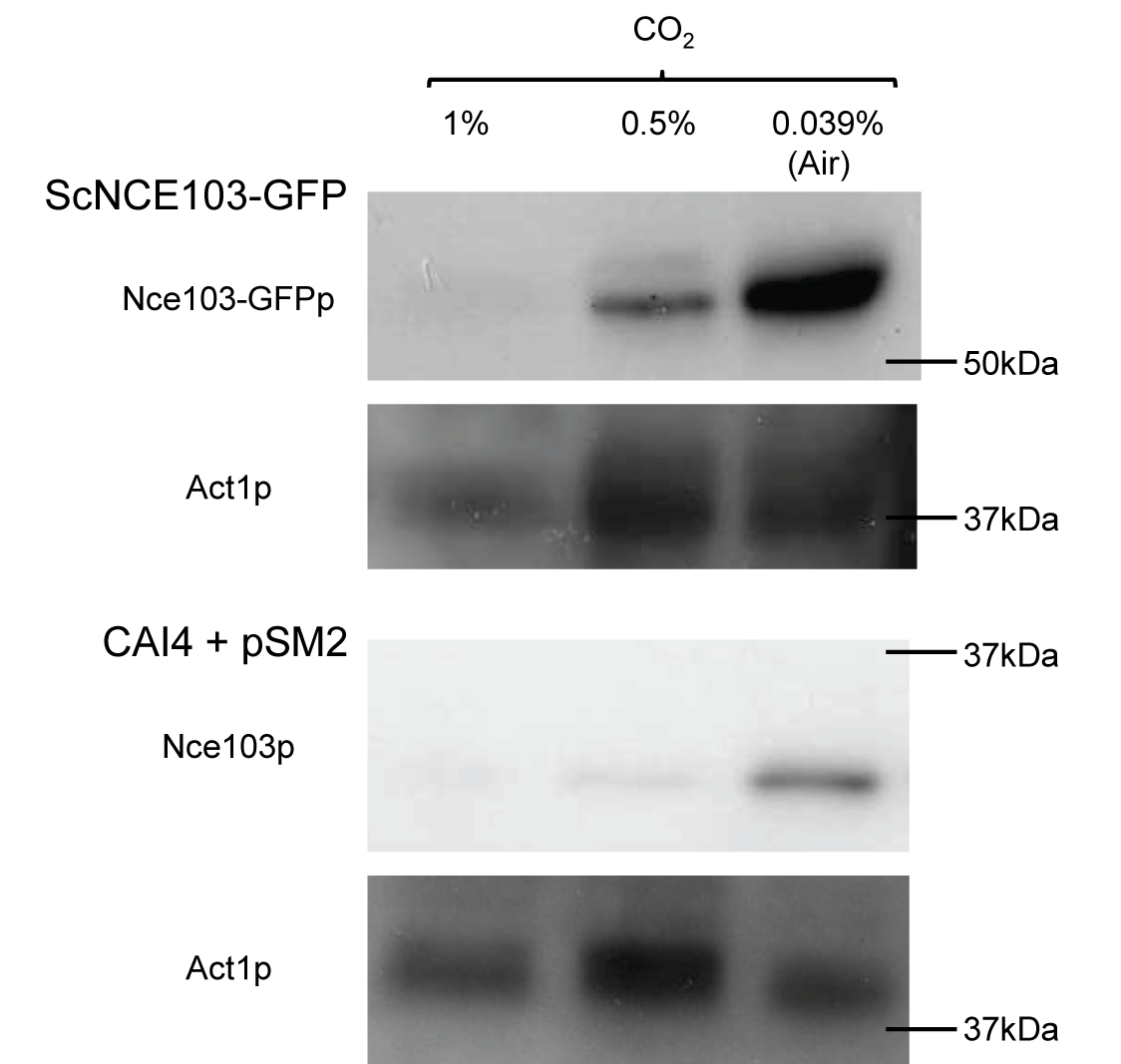

Supplement: Figure S8 — Nce103p induction is exquisitely sensitive to ambient CO2 availability. Using appropriate antibodies, carbonic anhydrase signals are shown in western blots from S. cerevisiae (top) and C. albicans (bottom). Proteins were extracted from cells grown in YPD for 4h in air or air enriched with 0.5 or 1% CO2. Nce103p signals are detectable in ambient air, and air enriched with 0.5% CO2. (TIF) [file ppat.1002485.s008.tif]
