# Supplementary material for: The bZIP Transcription Factor Rca1p Is a Central Regulator of a Novel CO2 Sensing Pathway in Yeast
Source: PLoS Pathog. 2012 Jan 12;8(1):e1002485. doi: 10.1371/journal.ppat.1002485 (PMC3257301; doi:10.1371/journal.ppat.1002485)
Supplement: Protocol S1 — Detailed protocols about media used in this study, strains and plasmids construction, yeast transformations, Southern blot analysis and the generation of C. albicans Nce103p antibodies, as well as supporting references. (DOC) [file ppat.1002485.s009.doc]

**Strains and media**

*C. albicans* and *S. cerevisiae* strains and transforming plasmids used in this study are listed in Table S3 and S4. Strains were grown in YPD (1% yeast extract, 2% Bacto peptone, and 2% glucose), and YNB (0.67% yeast nitrogen base and 2% glucose). Uridine (50 µg/ml), histidine (24 µg/ml), leucine (72 µg/ml) and methionine (24 µg/ml) were added to the YNB medium when needed. 10 mM dibutyryl cyclic AMP (dbcAMP; Sigma) was added to YPD medium when required. 5-FOA plate were used during the process of gene inactivation. *Escherichia coli* were grown in LB media (1% tryptone, 1% NaCl, 0.5% yeast extract). Media were solidified by addition of 2% agar.

**Strains construction**

The initial *RCA1* mutant of the transcription factor library was obtained by PCR-based gene targeting using the strain BWP17 and 120-mer oligos designed upstream and downstream of the *RCA1* ORF (ORF19-6102ATG; ORF19-6102STOP). Primer ORF19-6102ATG and ORF19-6102STOP were used for amplifying the *C. albicans ARG4* and *HIS1* genes cloned in pFA-ARG4 and pFA-HIS1 as described . *C. albicans RCA1* was then inactivated in CAI4 , using the *HisG-URA3-HisG* cassette to disrupt the 852 bp *RCA1* open reading frame (GenBank association number EAL00056) from positions +366 to +761. After passage on 5-FOA, the heterozygous strain (*rca1Δ*/*RCA1*) was used for a second round of transformation. The resulting mutants were exposed to 5-FOA and finally transformed with plasmid pSM2 to produce *rca1Δ* strains; pSM2-RCA1 to generate strain *rca1Δ+RCA1*, and pSM2-RCA1-HA_3_ for strain *rca1Δ+RCA1-HA_3_*, pSM2-RCA1-S124A for strain *rca1Δ+RCA1-*S124A, pSM2-RCA1-S126A for strain *rca1Δ+RCA1-*S126A, pSM2-RCA1-S222G for strain *rca1Δ+RCA1-*S222G (plasmids are described in the respective Plasmid section below). All plasmids were linearized by *Hpa*I and integrated at the *URA3* locus. Correct integrations were confirmed by Southern Blot (Figure S2). *RCA1* partially overlaps two genes (*orf19.6103* and *MVD*); inactivation was specifically designed to not alter the expression of the two genes. qRT-PCR confirmed that the expression of both genes remained unchanged in the *rca1Δ* mutant (Figure S3).

In *S. cerevisiae*, full length Sc*NCE103* (GenBank association number DAA10509.1) was inactivated in a BY4741 background by amplification of a *KanMX* cassette from plasmid pUG6 with primers Nce.Ko.Kan-F and Nce.Ko.Kan-R. This produced strain Sc*nce103Δ*. Full length *CST6* (GenBank association number DAA08512.1) was inactivated in a ScNCE103-GFP background (generated and verified by Invitrogen). The *CST6* disruption cassette was produced by amplification of the *URA3* or KAN cassette from pUG72 or pUG6 with primers ScCST6.Ko.Kan-F and ScCST6.Ko.Kan-R. This generated strain ScNCE103-GFP+*cst6Δ* and ScNCE103-GFP+*cst6ΔKan*. Correct inactivations were confirmed by diagnostic PCR or qRT-PCR (Figure S3). BY4741+pTEF-GFP strain carrying GFP under the control of *TEF* promoter integrated instead of *URA3* gene was constructed by transforming the cells with DNA cassette generated by PCR using the primers FwGP and RvGP and plasmid pYM-N21 .

**Yeast Transformations**

Yeast transformations were performed by the lithium acetate-PEG protocol for *C. albicans* and *S. cerevisiae* .

**Plasmid construction**

All primers used in this study are listed in Table S5. Plasmid pRCA1.KO.URAb, used for the inactivation of *RCA1,* was obtained by introduction of two *RCA1* sequences: one 616bp fragment (amplified with primers RCA1-F-SacI and RCA1-R-BglII using genomic DNA from *C. albicans* SC5314 as template) and a 629 bp fragment (RCA1-F-BamHI and RCA1-R-HindIII) subsequently cloned to flank the *URA3*-blaster on plasmid pURAb . Digestion of this plasmid by *Sac*I liberates the cassette used for *RCA1* deletion.

Complementation of the *CaNCE103* mutant was achieved by introduction of plasmid pSM2-NCE103, digested with *Hpa*I, at the *URA3* locus. For this purpose plasmid pSM2 was digested with *Xba*I and *Bam*HI and ligated to a 726bp fragment of *NCE103* including the promoter and open reading frame (amplified with primers pMB5-F and pMB5–R). This plasmid was used to transform the Ca*nce103∆* strain TK1 generating Ca*nce103∆*+pSM2-NCE103. As a control plasmid pSM2 was used to transform TK1 to generate Ca*nce103Δ*. Plasmid pSM2-RCA1 was obtained by digesting pSM2 with *BamH*I and *Not*I, and cloning a 2.3 kbp fragment containing the promoter and ORF of *RCA1*, which was amplified from genomic DNA using primers Orf19.6102-F2 and Orf19.6102-R2 and with *Bgl*II and *Not*I. HA tagging of *RCA1* was achieved by initially amplifying a 1.8 kbp *RCA1* fragment (using primers Orf19.6102-F2 and Orf19.6102(HA)) which was subsequently ligated into pFM-2 by *Bgl*II and *Kpn*I digestion. Then a HA_3_ tag from plasmid pMPY-3×HA was integrated via *Not*I digestion as described in generating pFM2-RCA1-HA3. Subsequently *RCA1-HA_3_* was amplified from pFM2-RCA1-HA3 with primers Orf19.6102-F2 and Orf19.6102-HA-R2, digested with *Bgl*II, and integrated into pSM2 which was cut with *Bam*HI to create pSM2-RCA1-HA_3_. The latter and pSM2-RCA1 were linearised by *Hpa*I and transformed into *rca1Δ*/*RCA1* and *rca1Δ*/*rca1Δ* to produce strains *rca1Δ*/*RCA1*+pSM2-RCA1-HA_3_ and *rca1Δ*+pSM2-RCA1-HA_3_; *rca1Δ*/*RCA1*+pSM2-RCA1-HA_3_ and *rca1Δ*+pSM2-RCA1-HA_3_ respectively. As a control plasmid pSM2 was used to transform the *rca1* homozygous mutant (*rca1Δ*/*rca1Δ*) to generate *rca1Δ*. Strains were validated by Southern blot (Figure S2).

Plasmids pSM2-RCA1-S124A, pSM2-RCA1-S126A and pSM2-RCA1-S222G were construct with the same strategy. As example, two fragments amplified by Orf19.6102-F2/S124A-R and S124A-F/Orf19.6102-HA-R2 using genomic DNA from *C. albicans* SC5314 served as template for a second PCR with primers Orf19.6102-F2 and Orf19.6102-HA-R2. This fragment was digested by *Not*I and *Bgl*II and ligate in pSM2 digested by *Not*I and *Bam*HI. The three resulting plasmids were digested by *Hpa*I to transform the *rca1* homozygous mutant (*rca1Δ*/*rca1Δ*) and generate respectively strain *rca1Δ+RCA1-*S124A, *rca1Δ+RCA1-*S126A and *rca1Δ+RCA1-*S222G.

Sc*NCE103*Δ complementation was possible by introduction of plasmid pScNCE103-GFP. This construct is the result of pRS316 digested by *Not*I and *Bam*HI and ligated to a fragment containing the *ScNCE103-GFP* fusion and 1kb of the Sc*NCE103* promoter (amplified by ScNCE-1 and ScNCE-end primer on ScNCE103-GFP (Invitrogen) genomic DNA), previously cut by *Not*I and *Bam*HI. Mutated *S. cerevisiae* *NCE103* promoter, pScNCE103-GFP-MUT, was obtained after digestion of pScNCE103-GFP with *Aat*II, blunting with Klenow fragment (Fermentas) and re-ligation. Sequencing confirmed the mutation of the original “gtTGACGTCAga” sequence present in position -285 from the ATG to “gtTGCAga”. Episomal plasmids pScNCE103-GFP and pScNCE103-GFP-MUT were introduced in Sc*nce103Δ* to create respectively Sc*nce103Δ+*pScNCE103-GFP and ScNCE103-GFP-MUT. pRS316-CST6 is the result of pRS316 digested by *Not*I and *Hind*III and ligated to a fragment containing the *CST6* ORF and 1kb of its promoter (amplified by CST6-F and CST6-R primers on BY4741 genomic DNA), previously cut with the same restriction enzyme.

**Southern blot analysis**

*C. albicans* *RCA1* inactivation was confirmed by Southern blot analysis using DIG High primer DNA labeling and detection as per the manufacture’s recommendations (Roche). The DNA *RCA1* probe (0.6kb) was PCR amplified using primers RCA1-F-SacI and RCA1-R-BglII on *C. albicans* SC5314 genomic DNA as template.

**Generation of *C. albicans* Nce103p antibodies**

The *C. albicans* *NCE103* ORF was amplified by primers CaNCE-FVamGEX/NCE-BR and integrated into pCR2.1 TOPO (Invitrogen) to obtain pCR2.1 BamNCE. The latter was digested with *Bam*HI and *Eco*RI to integrate Ca*NCE103* behind the glutathione S-transferase (GST) gene of the expression plasmid pGEX-6P-2 (GE Healthcare). The resulting plasmid, pGEX-6P-2-NCE103, was transformed into *E. coli* BL21(DE3) (Invitrogen). Induction of the GST-Nce103p expression was realised by addition of 0.2 mM IPTG (Melford) to LB media with 50 µg/ml ampicillin (Melford) and incubated for 4 hrs. After induction, cells were harvested at 3000 rpm, 10 min, 4º C and the pellet was resuspended in 1x PBS buffer (140 mM NaCl, 2.7 mM KCl, 10 mM Na_2_HPO_4_ and 1.8 mM KH_2_PO_4_, pH 7.3). To prevent degradation, 100 mM PMSF and 1 tablet of protease inhibitor cocktail (Roche) was added to the suspension. The cell suspension was sonicated for 10 min. (15 sec x 10, with 45 sec on ice in between) and subsequently centrifuged at 3.000 rpm, 15 minutes, 4º C. The supernatant was used for protein purification. Glutathione Sepharose 4B was used for column purification of recombinant GST-Nce103p fusion. The column was prepared according to manufacturer’s instructions (GE Healthcare). The GST column was equilibrated with 5 washes of 1x PBS buffer. After application of cells extract, the column was subsequently washed with 1x PBS buffer. The tip of the column was covered and elution buffer (10 mM Glutathione and 50 mM Tris) was added. After 10 min incubation, the protein sample was collected in an Eppendorf tube. The elution step was repeated 5 times. The protein samples were stored at –20˚C , and subsequently used to immunized rabbits (Harlan Sera-Lab). After the final test bleed polyclonal antibodies against *C. albicans* Nce103p were obtained and used for western blot analysis as described below.

**Supporting references**

1. Boeke JD, LaCroute F, Fink GR (1984) A positive selection for mutants lacking orotidine-5'-phosphate decarboxylase activity in yeast: 5-fluoro-orotic acid resistance. Mol Gen Genet 197: 345-346.

2. Gola S, Martin R, Walther A, Dunkler A, Wendland J (2003) New modules for PCR-based gene targeting in Candida albicans: rapid and efficient gene targeting using 100 bp of flanking homology region. Yeast 20: 1339-1347.

3. Fonzi WA, Irwin MY (1993) Isogenic strain construction and gene mapping in Candida albicans. Genetics 134: 717-728.

4. El Barkani A, Kurzai O, Fonzi WA, Ramon A, Porta A, et al. (2000) Dominant active alleles of RIM101 (PRR2) bypass the pH restriction on filamentation of Candida albicans. Mol Cell Biol 20: 4635-4647.

5. Guldener U, Heck S, Fielder T, Beinhauer J, Hegemann JH (1996) A new efficient gene disruption cassette for repeated use in budding yeast. Nucleic Acids Res 24: 2519-2524.

6. Gueldener U, Heinisch J, Koehler GJ, Voss D, Hegemann JH (2002) A second set of loxP marker cassettes for Cre-mediated multiple gene knockouts in budding yeast. Nucleic Acids Res 30: e23.

7. Janke C, Magiera MM, Rathfelder N, Taxis C, Reber S, et al. (2004) A versatile toolbox for PCR-based tagging of yeast genes: new fluorescent proteins, more markers and promoter substitution cassettes. Yeast 21: 947-962.

8. Walther A, Wendland J (2003) An improved transformation protocol for the human fungal pathogen Candida albicans. Curr Genet 42: 339-343.

9. Klengel T, Liang WJ, Chaloupka J, Ruoff C, Schroppel K, et al. (2005) Fungal adenylyl cyclase integrates CO2 sensing with cAMP signaling and virulence. Curr Biol 15: 2021-2026.

10. Muhlschlegel FA, Fonzi WA (1997) PHR2 of Candida albicans encodes a functional homolog of the pH-regulated gene PHR1 with an inverted pattern of pH-dependent expression. Mol Cell Biol 17: 5960-5967.

11. Schneider BL, Seufert W, Steiner B, Yang QH, Futcher AB (1995) Use of polymerase chain reaction epitope tagging for protein tagging in Saccharomyces cerevisiae. Yeast 11: 1265-1274.

12. Znaidi S, Barker KS, Weber S, Alarco AM, Liu TT, et al. (2009) Identification of the Candida albicans Cap1p regulon. Eukaryot Cell 8: 806-820.

13. Sikorski RS, Hieter P (1989) A system of shuttle vectors and yeast host strains designed for efficient manipulation of DNA in Saccharomyces cerevisiae. Genetics 122: 19-27.

14. Sambrook J, Fritsch, E. F., and Maniatis, T. (1989) Preparation and transformation of competent E coli.: Cold Spring Harbor Laboratory Press. 74-71.84 p.
