# Supplementary material for: The bZIP Transcription Factor Rca1p Is a Central Regulator of a Novel CO2 Sensing Pathway in Yeast
Source: PLoS Pathog. 2012 Jan 12;8(1):e1002485. doi: 10.1371/journal.ppat.1002485 (PMC3257301; doi:10.1371/journal.ppat.1002485)
Supplement: Table S1 — Rca1p-HA3 binding in air dataset. The following criteria were used: Log2 pseudo-median signal intensity threshold of ≥0.5 and p-value cut-off of ≤0.01 [12]. Contig19#: The Contig19 number on which a given binding peak is detected using the Tilescope software [12]. Location: Position of the binding peak in the corresponding Contig19 DNA sequence. Log2 pseudo-median signal intensity: Log2-transformed pseudo-median signal intensity of Rca1p-HA3-binding at the corresponding location. Target: orf19 nomenclature according to the C. albicans Assembly 19 of Rca1p-HA3 target gene, based on the location of the locus relative to the binding peak. Absence of information indicates that binding peaks are not clearly associated with promoter of ORFs. If the peak was found in the promoter region common to two adjacent ORFs, the two possible predicted target genes are shown, separated by “and”. CGD Gene name: Gene name of the corresponding target gene according to the Candida Genome Database (CGD) (www.candidagenome.org). Description: Gene description according to CGD. (DOCX) [file ppat.1002485.s010.docx]

| **Contig 19#** | **Location** | **Log2 pseudo-median signal intensity** | **Target** | **CGD Gene name** | **Description** |
| --- | --- | --- | --- | --- | --- |
| Contig19-10194 | 4621..5701 | 1.438 | orf19.3895 | *CHT2* | Chitinase, required for normal filamentous growth; mRNA binds to She3p and is localized to buds of yeast-form cells and hyphal tips; downregulated in core caspofungin response; induced in yeast-form cells; Cyr1p-, Efg1p-, pH-regulated |
| Contig19-10194 | 5821..6181 | 1.281 | orf19.3897 |  | Predicted ORF in Assemblies 19, 20 and 21; decreased transcription is observed upon fluphenazine treatment or in an azole-resistant strain that overexpresses CDR1 and CDR2; transcription is repressed in response to alpha pheromone in SpiderM medium |
| Contig19-10194 | 57181..57421 | 1.263 | orf19.3928 and orf19.3929 |  | Putative transcription factor with zinc finger DNA-binding motif |
| Contig19-10198 | 17881..18361 | 1.184 |  |  |  |
| Contig19-2513 | 118741..119221 | 1.182 | orf19.7391 | *OCH1* | alpha-1,6-mannosyltransferase; initiates N-glycan outer chain branch addition; similar to S. cerevisiae Och1p; required for wild-type virulence in mouse intravenous infection; fungal-specific (no human or murine homolog) |
| Contig19-10186 | 57721..58021 | 1.128 |  |  |  |
| Contig19-10202 | 208561..209221 | 1.119 | orf19.4336 | *RPS5* | Predicted ribosomal protein; macrophage/pseudohyphal-induced after 16 h; genes encoding cytoplasmic ribosomal subunits, translation factors, and tRNA synthetases are downregulated upon phagocytosis by murine macrophage |
| Contig19-2500 | 98521..99061 | 1.021 |  |  |  |
| Contig19-10198 | 1..841 | 1.013 |  |  |  |
| Contig19-10105 | 16441..16801 | 1.005 |  |  |  |
| Contig19-10170 | 40021..40261 | 1.004 | orf19.3178 |  |  |
| Contig19-10170 | 46321..46561 | 1.002 |  |  |  |
| Contig19-10202 | 61741..62161 | 0.997 | orf19.4250 and orf19.4251 | 2) *ZCF22* | 2) Predicted zinc-finger protein of unknown function |
| Contig19-10198 | 2821..3001 | 0.961 | orf19.4132 |  |  |
| Contig19-10093 | 22381..22561 | 0.958 | orf19.1117 |  | Predicted ORF in Assemblies 19, 20 and 21; similar to Candida boidinii formate dehydrogenase; virulence-group-correlated expression |
| Contig19-10202 | 60421..61021 | 0.951 |  |  |  |
| Contig19-10170 | 66061..66661 | 0.943 | orf19.3195 | *HIP1* | Alkaline upregulated; flucytosine induced; regulated by Plc1p, Gcn2p and Gcn4p; fungal-specific (no human or murine homolog) |
| Contig19-10170 | 44101..44281 | 0.937 | orf19.3183 and orf19.3184 |  |  |
| Contig19-10198 | 1561..1981 | 0.935 |  |  |  |
| Contig19-10202 | 221941..222301 | 0.924 |  |  |  |
| Contig19-10234 | 54241..56161 | 0.922 | orf19.5808 |  |  |
| Contig19-10212 | 274741..275041 | 0.915 |  |  |  |
| Contig19-10137 | 52801..53221 | 0.906 | orf19.1957 | *CYC3* | Cytochrome c heme lyase, mitochondrial; gene also encodes antigenic cell-wall protein; mRNA more abundant in filaments than yeast-form; induced on polystyrene adherence, interaction with macrophage; N-glycosylation, 2 heme-binding motifs |
| Contig19-2449 | 26041..26641 | 0.900 |  |  |  |
| Contig19-2456 | 721..1921 | 0.899 | orf19.6614 |  | Deleted in assembly 21 |
| Contig19-10202 | 56281..56641 | 0.878 | orf19.4246 |  | Protein with similarity to S. cerevisiae Ykr070wp; transposon mutation affects filamentous growth; Hog1p-downregulated; shows colony morphology-related gene regulation by Ssn6p; induced during cell wall regeneration; possibly essential gene |
| Contig19-2511 | 38221..38941 | 0.872 | orf19.7302 |  |  |
| Contig19-10247 | 80821..82441 | 0.869 |  |  |  |
| Contig19-10192 | 117961..119701 | 0.866 |  |  |  |
| Contig19-10119 | 95281..95701 | 0.865 |  |  |  |
| Contig19-10137 | 11281..11521 | 0.862 | orf19.1932 | *CFL4* | Similar to ferric reductase, C-terminal region; expression greater in low iron; transcription is negatively regulated by Sfu1p; ciclopirox olamine induced; shows colony morphology-related gene regulation by Ssn6p |
| Contig19-2511 | 128581..128761 | 0.857 |  |  |  |
| Contig19-10202 | 109261..109561 | 0.840 |  |  |  |
| Contig19-10212 | 68941..70621 | 0.838 | orf19.4597 |  | Predicted ORF in Assemblies 19, 20 and 21; possibly an essential gene, disruptants not obtained by UAU1 method |
| Contig19-1876 | 5341..5941 | 0.835 |  |  |  |
| Contig19-10202 | 14941..15241 | 0.834 | orf19.4227 |  |  |
| Contig19-10176 | 74401..74821 | 0.828 |  |  |  |
| Contig19-10238 | 37501..39901 | 0.827 |  |  |  |
| Contig19-10216 | 175801..176161 | 0.820 |  |  |  |
| Contig19-10104 | 4861..5341 | 0.817 |  |  |  |
| Contig19-10104 | 99241..101341 | 0.812 |  |  |  |
| Contig19-10203 | 80041..81901 | 0.812 | orf19.4388 |  | Predicted ORF in Assemblies 19, 20 and 21; has histone fold domain; similar to TAFII47 proteins from S. cerevisiae, Danio rerio, Drosophila melanogaster, human, and mouse |
| Contig19-10137 | 59641..59941 | 0.811 | orf19.1958 |  |  |
| Contig19-10080 | 21421..21601 | 0.803 |  |  |  |
| Contig19-10184 | 47881..48181 | 0.803 |  |  |  |
| Contig19-10202 | 152281..152581 | 0.802 |  |  |  |
| Contig19-10080 | 20761..21121 | 0.795 |  |  |  |
| Contig19-10132 | 10441..10861 | 0.793 |  |  |  |
| Contig19-10203 | 109081..109450 | 0.791 | orf19.4401 | *YVH1* | Putative dual specificity phosphatase (phosphoserine/threonine and phosphotyrosine phosphatase); similar to S. cerevisiae Yvh1p; required for wild-type growth rate and for wild-type virulence in mouse model of systemic infection |
| Contig19-10227 | 152881..153531 | 0.783 | orf19.5475 |  |  |
| Contig19-10206 | 39181..40261 | 0.778 |  |  |  |
| Contig19-1859 | 8101..8530 | 0.776 |  |  |  |
| Contig19-10065 | 18721..19321 | 0.771 |  |  |  |
| Contig19-10104 | 361..901 | 0.770 | orf19.1227 | *ZCF4* | Putative transcription factor with zinc cluster DNA-binding motif; possibly spurious ORF (Annotation Working Group prediction) |
| Contig19-10198 | 16981..17341 | 0.766 | orf19.4144 |  | Predicted ORF in Assemblies 19, 20 and 21; clade-associated gene expression |
| Contig19-10194 | 24001..24241 | 0.765 |  |  |  |
| Contig19-10216 | 110341..110761 | 0.765 | orf19.4956 | *RPN1* | Predicted ORF in Assemblies 19, 20 and 21; regulated by Gcn2p and Gcn4p |
| Contig19-1086 | 421..961 | 0.762 |  |  |  |
| Contig19-10123 | 1..481 | 0.760 |  |  |  |
| Contig19-10119 | 192541..192721 | 0.751 |  |  |  |
| Contig19-10090 | 27961..28441 | 0.750 |  |  |  |
| Contig19-10123 | 56221..56401 | 0.737 | orf19.1630 |  |  |
| Contig19-10237 | 76081..76321 | 0.736 |  |  |  |
| Contig19-2456 | 12061..13021 | 0.733 |  |  |  |
| Contig19-10202 | 102241..102541 | 0.732 | orf19.4271 |  |  |
| Contig19-10212 | 123061..123361 | 0.729 |  |  |  |
| Contig19-10233 | 481..961 | 0.725 |  |  |  |
| Contig19-10198 | 40081..40441 | 0.721 |  |  |  |
| Contig19-10194 | 215221..215521 | 0.720 | orf19.4007 |  |  |
| Contig19-1914 | 661..1501 | 0.720 |  |  |  |
| Contig19-10202 | 218761..219061 | 0.710 |  |  |  |
| Contig19-10126 | 12601..13081 | 0.709 |  |  |  |
| Contig19-10202 | 223381..223681 | 0.708 | orf19.4342 |  |  |
| Contig19-2500 | 2401..2641 | 0.707 |  |  |  |
| Contig19-10123 | 238201..238681 | 0.706 |  |  |  |
| Contig19-10202 | 39901..40441 | 0.704 |  |  |  |
| Contig19-10202 | 220981..221401 | 0.704 |  |  |  |
| Contig19-10158 | 210901..211081 | 0.701 |  |  |  |
| Contig19-10170 | 85201..85681 | 0.701 |  |  |  |
| Contig19-10227 | 150121..152281 | 0.700 | orf19.5474 and orf19.5475 |  | 1) Predicted ORF in Assemblies 19, 20 and 21; possibly spurious ORF (Annotation Working Group prediction); transcriptionally activated by Mnl1p under weak acid stress |
| Contig19-10202 | 165841..166741 | 0.695 |  |  |  |
| Contig19-2456 | 9061..11221 | 0.695 |  |  |  |
| Contig19-10065 | 19621..20101 | 0.692 |  |  |  |
| Contig19-10087 | 43441..44161 | 0.692 |  |  |  |
| Contig19-10176 | 95881..96241 | 0.692 | orf19.3435 |  |  |
| Contig19-10202 | 244441..244621 | 0.692 |  |  |  |
| Contig19-10123 | 217201..217921 | 0.688 | orf19.1707 |  |  |
| Contig19-10123 | 8341..8641 | 0.687 | orf19.1608 |  |  |
| Contig19-10220 | 10261..10441 | 0.678 |  |  |  |
| Contig19-10198 | 7261..7501 | 0.677 |  |  |  |
| Contig19-10198 | 23521..23941 | 0.677 |  |  |  |
| Contig19-10070 | 10081..10681 | 0.673 |  |  |  |
| Contig19-10202 | 192181..192601 | 0.668 |  |  |  |
| Contig19-10202 | 219661..220081 | 0.668 |  |  |  |
| Contig19-10233 | 98341..99541 | 0.667 | orf19.5752 |  |  |
| Contig19-10155 | 28561..28801 | 0.663 | orf19.2649 | *PCL1* | Cyclin homolog; expression induced upon filamentous growth; transcription is induced in response to alpha pheromone in SpiderM medium |
| Contig19-10202 | 39061..39241 | 0.660 |  |  |  |
| Contig19-10137 | 44941..45241 | 0.657 | orf19.1950 |  |  |
| Contig19-10225 | 30421..30841 | 0.655 |  |  |  |
| Contig19-10063 | 12841..13741 | 0.653 | orf19.639 |  | Predicted ORF in Assemblies 19, 20 and 21; decreased transcription is observed upon fluphenazine treatment or in an azole-resistant strain that overexpresses CDR1 and CDR2 |
| Contig19-10070 | 32341..32581 | 0.648 |  |  |  |
| Contig19-10236 | 309181..309541 | 0.648 |  |  |  |
| Contig19-2134 | 5401..7321 | 0.647 |  |  |  |
| Contig19-10162 | 68161..68761 | 0.646 |  |  |  |
| Contig19-10227 | 141901..142981 | 0.644 |  |  |  |
| Contig19-10237 | 194161..194941 | 0.641 | orf19.6114 |  |  |
| Contig19-10057 | 1201..1441 | 0.638 | orf19.567 | *TFB3* | Putative transcription factor with C3HC4 zinc finger DNA-binding motif; transcription is positively regulated by Tbf1p |
| Contig19-10186 | 34801..36241 | 0.638 | orf19.3673 |  | In S.c. TRS23 One of 10 subunits of the transport protein particle (TRAPP) complex of the cis-Golgi which mediates vesicle docking and fusion; involved in endoplasmic reticulum (ER) to Golgi membrane traffic; human homolog is TRAPPC4 |
| Contig19-2500 | 80041..81121 | 0.638 |  |  |  |
| Contig19-10150 | 45361..45661 | 0.637 |  |  |  |
| Contig19-10127 | 9241..9481 | 0.634 |  |  |  |
| Contig19-10202 | 54781..55081 | 0.633 | orf19.4245 |  |  |
| Contig19-10035 | 61141..61441 | 0.631 |  |  |  |
| Contig19-10123 | 248941..249121 | 0.629 | orf19.1721 and orf19.1720 | 1) *NCE103* | 1) Carbonic anhydrase involved in the conversion of carbon dioxide to bicarbonate; essential for pathogenesis in host niches with limited CO2, wild-type white-opaque switching; biofilm induced; activated by Mnl1p under weak acid stress |
| Contig19-10225 | 142741..143701 | 0.628 |  |  |  |
| Contig19-10137 | 108001..108241 | 0.626 |  |  |  |
| Contig19-1859 | 6901..7501 | 0.625 |  |  |  |
| Contig19-10230 | 162601..163741 | 0.623 |  |  |  |
| Contig19-10212 | 147661..147961 | 0.622 | orf19.4641 | *NMT1* | Myristoyl-CoA:protein N-myristoyltransferase; attaches the fatty acid myristate to a small number of proteins at an N-terminal Gly; essential; antifungal drug target; functional homolog of S. cerevisiae Nmt1p |
| Contig19-10170 | 26521..26821 | 0.621 | orf19.3172 and orf19.3173 |  |  |
| Contig19-10202 | 108481..108781 | 0.621 |  |  |  |
| Contig19-10185 | 20881..21361 | 0.614 |  |  |  |
| Contig19-10201 | 30421..32221 | 0.607 |  |  |  |
| Contig19-10198 | 4981..5221 | 0.605 |  |  |  |
| Contig19-1127 | 1861..2050 | 0.604 |  |  |  |
| Contig19-10194 | 266941..268021 | 0.602 | orf19.4054 | *CTA24* | Putative transcriptional activator, downregulated by Efg1p; member of a family of telomere-proximal genes; transcription is upregulated in an RHE model of oral candidiasis |
| Contig19-10215 | 113161..113641 | 0.601 |  |  |  |
| Contig19-10171 | 7321..7681 | 0.600 |  |  |  |
| Contig19-10140 | 56461..57061 | 0.599 |  |  |  |
| Contig19-2405 | 28861..29041 | 0.598 | orf19.6192 |  | Predicted ORF in Assemblies 19, 20 and 21; Plc1p-regulated; possibly spurious ORF |
| Contig19-2413 | 17761..18661 | 0.598 |  |  |  |
| Contig19-1876 | 1081..1381 | 0.597 |  |  |  |
| Contig19-10090 | 30181..30601 | 0.594 |  |  |  |
| Contig19-10171 | 28021..28261 | 0.587 | orf19.3219 |  |  |
| Contig19-10212 | 50581..51121 | 0.587 |  |  |  |
| Contig19-10225 | 32461..33241 | 0.584 | orf19.5306 |  |  |
| Contig19-1859 | 5521..6781 | 0.583 |  |  |  |
| Contig19-10241 | 65281..66481 | 0.580 | orf19.6227 |  |  |
| Contig19-10237 | 191581..192541 | 0.579 |  |  |  |
| Contig19-10237 | 196201..196621 | 0.579 | orf19.6115 |  |  |
| Contig19-2506 | 1141..2101 | 0.578 |  |  |  |
| Contig19-2506 | 4501..5281 | 0.578 |  |  |  |
| Contig19-2506 | 2761..3001 | 0.577 |  |  |  |
| Contig19-10166 | 2761..4081 | 0.573 |  |  |  |
| Contig19-1472 | 1741..2101 | 0.571 |  |  |  |
| Contig19-10136 | 16081..17161 | 0.570 | orf19.1825 |  | Protein not essential for viability; filament induced; regulated by Nrg1p, Rfg1p, Tup1p |
| Contig19-10139 | 124921..125281 | 0.570 | orf19.2061 |  |  |
| Contig19-2516 | 164701..165841 | 0.570 |  |  |  |
| Contig19-1888 | 3541..4441 | 0.568 |  |  |  |
| Contig19-2518 | 136501..137101 | 0.568 |  |  |  |
| Contig19-10014 | 33421..33661 | 0.567 |  |  |  |
| Contig19-10202 | 13261..13561 | 0.567 |  |  |  |
| Contig19-10237 | 198781..199081 | 0.566 |  |  |  |
| Contig19-10170 | 17881..18061 | 0.559 | orf19.3170 |  |  |
| Contig19-10170 | 99961..100400 | 0.558 |  |  |  |
| Contig19-10186 | 59461..59881 | 0.558 | orf19.3683 | *AGE3* | Putative ADP-ribosylation factor GTPase activating protein, functional ortholog of S. cerevisiae GCS1; mutation affects endocytosis, hyphal growth, chemical and drug resistance, and sensitivity to cell wall inhibitors |
| Contig19-10194 | 269461..269701 | 0.558 |  |  |  |
| Contig19-2500 | 30601..31081 | 0.556 |  |  |  |
| Contig19-10209 | 58321..58681 | 0.555 |  |  |  |
| Contig19-2134 | 4681..5221 | 0.555 |  |  |  |
| Contig19-10131 | 6061..6361 | 0.554 |  |  |  |
| Contig19-10131 | 8461..8641 | 0.554 |  |  |  |
| Contig19-1996 | 2221..2881 | 0.554 |  |  |  |
| Contig19-10237 | 197521..198121 | 0.552 |  |  |  |
| Contig19-10194 | 270121..270541 | 0.551 |  |  |  |
| Contig19-2456 | 13201..13450 | 0.549 |  |  |  |
| Contig19-1472 | 2641..2890 | 0.548 |  |  |  |
| Contig19-10057 | 7021..7621 | 0.547 |  |  |  |
| Contig19-2516 | 167101..167410 | 0.545 |  |  |  |
| Contig19-10202 | 224641..224941 | 0.543 |  |  |  |
| Contig19-10215 | 80341..80581 | 0.543 |  |  |  |
| Contig19-10057 | 34861..35341 | 0.542 |  |  |  |
| Contig19-2507 | 51961..53041 | 0.537 |  |  |  |
| Contig19-10228 | 38221..38401 | 0.534 |  |  |  |
| Contig19-10254 | 98581..98761 | 0.532 | orf19.9773 |  |  |
| Contig19-10202 | 31741..31921 | 0.523 |  |  |  |
| Contig19-2134 | 2821..3001 | 0.521 |  |  |  |
| Contig19-2201 | 16981..17161 | 0.517 |  |  |  |
| Contig19-10202 | 60061..60301 | 0.509 |  |  |  |
| Contig19-10072 | 15061..15301 | 0.508 | orf19.741 |  |  |
| Contig19-10194 | 258061..258361 | 0.502 | orf19.4043 |  |  |
| Contig19-10194 | 95641..95821 | 0.460 | orf19.3952 |  |  |
