# Supplementary material for: The bZIP Transcription Factor Rca1p Is a Central Regulator of a Novel CO2 Sensing Pathway in Yeast
Source: PLoS Pathog. 2012 Jan 12;8(1):e1002485. doi: 10.1371/journal.ppat.1002485 (PMC3257301; doi:10.1371/journal.ppat.1002485)
Supplement: Table S2 — Rca1p-HA3 binding in 5.5% CO2 dataset. The following criteria were used: Log2 pseudo-median signal intensity threshold of ≥0.5 and p-value cut-off of ≤0.01 [12]. Contig19#: The Contig19 number on which a given binding peak is detected using the Tilescope software [12]. Location: Position of the binding peak in the corresponding Contig19 DNA sequence. Log2 pseudo-median signal intensity: Log2-transformed pseudo-median signal intensity of Rca1p-HA3-binding at the corresponding location. Target: orf19 nomenclature according to the C. albicans Assembly 19 of Rca1p-HA3 target gene, based on the location of the locus relative to the binding peak. Absence of information indicates that binding peaks are not clearly associated with promoter of ORFs. If the peak was found in the promoter region common to two adjacent ORFs, the two possible predicted target genes are shown, separated by “and”. CGD Gene name: Gene name of the corresponding target gene according to the Candida Genome Database (CGD) (www.candidagenome.org). Description: Gene description according to CGD. (DOCX) [file ppat.1002485.s011.docx]

| **Contig 19#** | **Location** | **Log2 pseudo-median signal intensity** | **Target** | **CGD Gene name** | **Description** |
| --- | --- | --- | --- | --- | --- |
| Contig19-10194 | 4681..5581 | 1.262 | orf19.3895 | *CHT2* | Chitinase, required for normal filamentous growth; mRNA binds to She3p and is localized to buds of yeast-form cells and hyphal tips; downregulated in core caspofungin response; induced in yeast-form cells; Cyr1p-, Efg1p-, pH-regulated |
| Contig19-10194 | 5821..6181 | 1.107 | orf19.3897 |  | Predicted ORF in Assemblies 19, 20 and 21; decreased transcription is observed upon fluphenazine treatment or in an azole-resistant strain that overexpresses CDR1 and CDR2; transcription is repressed in response to alpha pheromone in SpiderM medium |
| Contig19-10170 | 46381..46561 | 0.977 |  |  |  |
| Contig19-2513 | 118681..119161 | 0.908 | orf19.7391 | *OCH1* | alpha-1,6-mannosyltransferase; initiates N-glycan outer chain branch addition; similar to S. cerevisiae Och1p; required for wild-type virulence in mouse intravenous infection; fungal-specific (no human or murine homolog) |
| Contig19-10104 | 4861..5281 | 0.883 |  |  |  |
| Contig19-10041 | 11221..11401 | 0.882 | orf19.177 |  |  |
| Contig19-10198 | 1..781 | 0.878 |  |  |  |
| Contig19-10186 | 57001..58141 | 0.871 |  |  |  |
| Contig19-10202 | 208621..209161 | 0.845 | orf19.4336 | *RPS5* | Predicted ribosomal protein; macrophage/pseudohyphal-induced after 16 h; genes encoding cytoplasmic ribosomal subunits, translation factors, and tRNA synthetases are downregulated upon phagocytosis by murine macrophage |
| Contig19-10198 | 17941..18301 | 0.815 | orf19.4144 |  | Predicted ORF in Assemblies 19, 20 and 21; clade-associated gene expression |
| Contig19-10119 | 95281..95521 | 0.808 |  |  |  |
| Contig19-10170 | 66061..66481 | 0.804 | orf19.3195 | *HIP1* | Alkaline upregulated; flucytosine induced; regulated by Plc1p, Gcn2p and Gcn4p; fungal-specific (no human or murine homolog) |
| Contig19-10123 | 8281..8701 | 0.796 | orf19.1608 |  |  |
| Contig19-10202 | 60541..61081 | 0.784 |  |  |  |
| Contig19-10105 | 16501..16801 | 0.778 |  |  |  |
| Contig19-10212 | 113281..113521 | 0.774 | orf19.4624 | *HRT2* | Protein described as having a role in Ty3 transposition; decreased expression in hyphae compared to yeast-form cells; protein detected by mass spec in stationary phase cultures |
| Contig19-10123 | 238201..238801 | 0.773 |  |  |  |
| Contig19-10198 | 1621..1981 | 0.77 |  |  |  |
| Contig19-2449 | 26101..26641 | 0.762 |  |  |  |
| Contig19-10202 | 61741..62041 | 0.759 | orf19.4250 and orf19.4251 | 2) *ZCF22* | 2) Predicted zinc-finger protein of unknown function |
| Contig19-2500 | 98581..99181 | 0.735 |  |  |  |
| Contig19-10215 | 113341..113641 | 0.728 |  |  |  |
| Contig19-10137 | 52861..53161 | 0.727 | orf19.1957 | *CYC3* | Cytochrome c heme lyase, mitochondrial; gene also encodes antigenic cell-wall protein; mRNA more abundant in filaments than yeast-form; induced on polystyrene adherence, interaction with macrophage; N-glycosylation, 2 heme-binding motifs |
| Contig19-10158 | 211801..212221 | 0.727 |  |  |  |
| Contig19-10037 | 7801..8161 | 0.718 | orf19.145 | *RPB4* | Protein similar to S. cerevisiae Rpb4p, which is a component of RNA polymerase II; transposon mutation affects filamentous growth |
| Contig19-10104 | 421..961 | 0.715 | orf19.1227 | *ZCF4* | Putative transcription factor with zinc cluster DNA-binding motif; possibly spurious ORF (Annotation Working Group prediction) |
| Contig19-10187 | 13861..14041 | 0.713 | orf19.3696 | *TOM22* | Predicted ORF in Assemblies 19, 20 and 21; shows colony morphology-related gene regulation by Ssn6p |
| Contig19-10219 | 28321..28561 | 0.713 |  |  |  |
| Contig19-10216 | 96661..96961 | 0.712 |  |  |  |
| Contig19-10125 | 1381..1741 | 0.71 |  |  |  |
| Contig19-10194 | 74401..75121 | 0.709 |  |  |  |
| Contig19-10216 | 110281..110881 | 0.701 | orf19.4956 | *RPN1* | Predicted ORF in Assemblies 19, 20 and 21; regulated by Gcn2p and Gcn4p |
| Contig19-10090 | 27901..28261 | 0.7 |  |  |  |
| Contig19-10202 | 221881..222241 | 0.699 |  |  |  |
| Contig19-2511 | 128581..128881 | 0.697 |  |  |  |
| Contig19-1086 | 301..1081 | 0.693 |  |  |  |
| Contig19-10137 | 11221..11581 | 0.689 | orf19.1932 | *CFL4* | Similar to ferric reductase, C-terminal region; expression greater in low iron; transcription is negatively regulated by Sfu1p; ciclopirox olamine induced; shows colony morphology-related gene regulation by Ssn6p |
| Contig19-10198 | 29101..29341 | 0.687 | orf19.4149 |  |  |
| Contig19-10212 | 281281..281521 | 0.686 |  |  |  |
| Contig19-10233 | 361..841 | 0.686 |  |  |  |
| Contig19-2507 | 33841..34201 | 0.684 |  |  |  |
| Contig19-10216 | 90901..91441 | 0.681 | orf19.4949 |  |  |
| Contig19-10202 | 218761..219181 | 0.678 |  |  |  |
| Contig19-10184 | 47701..48301 | 0.676 |  |  |  |
| Contig19-10176 | 74401..74881 | 0.675 |  |  |  |
| Contig19-10170 | 85021..85561 | 0.673 |  |  |  |
| Contig19-10123 | 181..601 | 0.671 |  |  |  |
| Contig19-10248 | 193141..193321 | 0.67 |  |  |  |
| Contig19-10121 | 361..661 | 0.668 |  |  |  |
| Contig19-10123 | 217201..217921 | 0.668 | orf19.1707 |  |  |
| Contig19-10125 | 70021..70321 | 0.665 |  |  |  |
| Contig19-10158 | 56221..56401 | 0.665 |  |  |  |
| Contig19-1193 | 1861..2331 | 0.665 |  |  |  |
| Contig19-10137 | 82981..83221 | 0.664 | orf19.1963 | *GDS1* |  |
| Contig19-10236 | 309001..309661 | 0.662 |  |  |  |
| Contig19-10073 | 481..661 | 0.655 |  |  |  |
| Contig19-1876 | 5521..5761 | 0.655 |  |  |  |
| Contig19-2456 | 1321..1861 | 0.653 | orf19.6614 |  | Deleted in assembly 21 |
| Contig19-10246 | 32221..32521 | 0.652 |  |  |  |
| Contig19-2500 | 49681..50041 | 0.652 |  |  |  |
| Contig19-10173 | 153361..153781 | 0.649 |  |  |  |
| Contig19-10126 | 25621..25921 | 0.648 |  |  |  |
| Contig19-10161 | 49081..49321 | 0.648 |  |  |  |
| Contig19-10150 | 153601..153841 | 0.647 | orf19.2511.1 | *MRLP33* | ORF predicted by Annotation Working Group, mito?; increased expression observed in an ssr1 homozygous null mutant; transcription is upregulated in both intermediate and mature biofilms |
| Contig19-10254 | 175201..175561 | 0.647 |  |  |  |
| Contig19-10150 | 45121..45781 | 0.643 |  |  |  |
| Contig19-10212 | 274741..275041 | 0.642 |  |  |  |
| Contig19-10063 | 32521..32701 | 0.641 |  |  |  |
| Contig19-2305 | 5521..5881 | 0.638 |  |  |  |
| Contig19-2511 | 38341..38641 | 0.638 | orf19.7302 |  |  |
| Contig19-2511 | 104821..105001 | 0.636 |  |  |  |
| Contig19-10035 | 61141..61441 | 0.634 |  |  |  |
| Contig19-10171 | 20281..20521 | 0.632 |  |  |  |
| Contig19-10063 | 541..781 | 0.628 |  |  |  |
| Contig19-10070 | 10201..10981 | 0.628 |  |  |  |
| Contig19-10065 | 18661..19921 | 0.627 |  |  |  |
| Contig19-10198 | 40021..40381 | 0.624 |  |  |  |
| Contig19-10208 | 47341..47581 | 0.623 | orf19.4511 |  |  |
| Contig19-10216 | 202201..202921 | 0.622 |  |  |  |
| Contig19-2479 | 2461..3601 | 0.622 | orf19.6834 |  | Deleted in assembly 21 |
| Contig19-2479 | 4021..4321 | 0.622 |  |  |  |
| Contig19-10202 | 104821..105241 | 0.619 |  |  |  |
| Contig19-10212 | 147781..148081 | 0.618 | orf19.4641 | *NMT1* | Myristoyl-CoA:protein N-myristoyltransferase; attaches the fatty acid myristate to a small number of proteins at an N-terminal Gly; essential; antifungal drug target; functional homolog of S. cerevisiae Nmt1p |
| Contig19-2449 | 15481..15841 | 0.618 |  |  |  |
| Contig19-10173 | 152101..152401 | 0.617 |  |  |  |
| Contig19-10247 | 81241..82681 | 0.616 |  |  |  |
| Contig19-2500 | 16801..17041 | 0.612 |  |  |  |
| Contig19-2500 | 49081..49381 | 0.612 |  |  |  |
| Contig19-10176 | 69241..69481 | 0.61 |  |  |  |
| Contig19-2201 | 16801..17240 | 0.61 |  |  |  |
| Contig19-10186 | 25201..26161 | 0.608 |  |  |  |
| Contig19-10218 | 27961..28201 | 0.605 | orf19.5065 |  |  |
| Contig19-10184 | 23341..23701 | 0.602 | orf19.3603 |  |  |
| Contig19-10151 | 19981..20281 | 0.599 | orf19.2530 and orf19.2532 | 2) *PRS* | 2) Putative prolyl-tRNA synthetase; monofunctional Class II synthetase; gene is constitutively expressed |
| Contig19-10246 | 17101..17521 | 0.598 |  |  |  |
| Contig19-10147 | 57721..58201 | 0.595 |  |  |  |
| Contig19-10104 | 99181..100381 | 0.593 |  |  |  |
| Contig19-10202 | 102301..102541 | 0.593 | orf19.4271 |  |  |
| Contig19-10170 | 99961..100450 | 0.588 |  |  |  |
| Contig19-10202 | 31441..31981 | 0.588 |  |  |  |
| Contig19-10170 | 78901..79261 | 0.585 |  |  |  |
| Contig19-10020 | 1..421 | 0.583 |  |  |  |
| Contig19-10202 | 219661..220021 | 0.582 |  |  |  |
| Contig19-10176 | 95881..96241 | 0.581 | orf19.3435 |  |  |
| Contig19-10215 | 66721..67801 | 0.577 |  |  |  |
| Contig19-10104 | 7501..8101 | 0.575 |  |  |  |
| Contig19-10159 | 1261..1561 | 0.575 |  |  |  |
| Contig19-10163 | 77761..78121 | 0.575 |  |  |  |
| Contig19-10202 | 173161..173521 | 0.575 |  |  |  |
| Contig19-10254 | 170461..170761 | 0.575 | orf19.6803 | *HUT1* |  |
| Contig19-10171 | 26941..27361 | 0.573 |  |  |  |
| Contig19-10248 | 1261..1741 | 0.572 |  |  |  |
| Contig19-10236 | 200641..200821 | 0.568 |  |  |  |
| Contig19-10126 | 12661..12901 | 0.567 |  |  |  |
| Contig19-10162 | 44641..44881 | 0.567 |  |  |  |
| Contig19-10202 | 224701..225061 | 0.564 |  |  |  |
| Contig19-10202 | 164761..165841 | 0.56 |  |  |  |
| Contig19-10218 | 30421..30661 | 0.559 |  |  |  |
| Contig19-10057 | 1261..1681 | 0.557 | orf19.567 | *TFB3* | Putative transcription factor with C3HC4 zinc finger DNA-binding motif; transcription is positively regulated by Tbf1p |
| Contig19-10216 | 201781..202081 | 0.557 |  |  |  |
| Contig19-10158 | 207541..207841 | 0.555 | orf19.2763 |  | Possibly transcriptionally regulated upon hyphal formation |
| Contig19-10170 | 94561..95161 | 0.553 |  |  |  |
| Contig19-10163 | 125581..126121 | 0.551 |  |  |  |
| Contig19-10147 | 59041..59461 | 0.549 | orf19.2374 and orf19.2375 |  | Deleted in assembly 21 |
| Contig19-10215 | 101941..102361 | 0.547 |  |  |  |
| Contig19-10230 | 214321..214621 | 0.547 | orf19.5630 | *APA2* | Protein described as ATP adenylyltransferase II; regulated by Gcn4p; repressed in response to amino acid starvation (3-aminotriazole treatment); increased expression in response to prostaglandins |
| Contig19-10170 | 82381..82621 | 0.544 |  |  |  |
| Contig19-10155 | 24361..24661 | 0.543 |  |  |  |
| Contig19-10090 | 30181..30601 | 0.542 |  |  |  |
| Contig19-10191 | 15781..15961 | 0.542 |  |  |  |
| Contig19-10227 | 153361..153531 | 0.534 | orf19.5475 |  |  |
| Contig19-10262 | 99721..99901 | 0.529 |  |  |  |
| Contig19-10248 | 1921..2101 | 0.527 |  |  |  |
| Contig19-2500 | 2401..2581 | 0.52 |  |  |  |
| Contig19-10073 | 1321..1681 | 0.518 |  |  |  |
| Contig19-10202 | 189301..189481 | 0.512 |  |  |  |
| Contig19-10170 | 85921..86221 | 0.509 |  |  |  |
| Contig19-10246 | 29161..29341 | 0.495 |  |  |  |
| Contig19-10170 | 98641..98821 | 0.448 |  |  |  |
| Contig19-10202 | 152401..152581 | 0.409 |  |  |  |
