# Supplementary material for: The bZIP Transcription Factor Rca1p Is a Central Regulator of a Novel CO2 Sensing Pathway in Yeast
Source: PLoS Pathog. 2012 Jan 12;8(1):e1002485. doi: 10.1371/journal.ppat.1002485 (PMC3257301; doi:10.1371/journal.ppat.1002485)
Supplement: Table S3 — Plasmids used and construct in this study. (DOCX) [file ppat.1002485.s012.docx]

| **Name** | **Parent** | **Target locus** | **Fragment** | **Reference** |
| --- | --- | --- | --- | --- |
| pSM2 |  | URA3 | *URA3* | ElBarkani et al., 2000 |
| pSM2-CaNCE103 | pSM2 | URA3 | *CaNCE103*; *URA3* | This work |
| pSM2-RCA1 | pSM2 | URA3 | *RCA1*; *URA3* | This work |
| pSM2-RCA1-HA_3_ | pSM2 | URA3 | *RCA1-HA3*; *URA3* | This work |
| pSM2-RCA1-S124A | pSM2 | URA3 | *RCA1-S124A*; *URA3* | This work |
| pSM2-RCA1-S126A | pSM2 | URA3 | *RCA1-S126A*; *URA3* | This work |
| pSM2-RCA1-S222G | pSM2 | URA3 | *RCA1-S222G*; *URA3* | This work |
| pFM-2 |  |  | *URA3* | Muhlschlegel and Fonzi, 1997 |
| pFM2-RCA1-HA_3_ | pFM-2 |  | *RCA1-HA3*; *URA3* | This work |
| pURAb |  |  | *HisG-URA3-HisG* | Fonzi and Irwin 1993 |
| pRCA1.KO.URAb | pURAb |  | *RCA1.KO.URAb* | This work |
| pMPY-3×HA |  |  | *HA_3_* | Schneider and et al., 1995 |
| pCR2.1 TOPO |  |  |  | Invitrogen |
| pCR2.1 BamNCE | pCR2.1 TOPO |  | Ca*NCE103* | This work |
| pGEX-6P-2 |  |  | *GST* | Amersham |
| pGEX-6P-2-*NCE103* | pGEX-6P-2 |  | *GST-CaNCE103* | This work |
| pRS316 |  | Auto-replicating | *URA3* | Sikorski and Hieter, 1989 |
| pRS316-CST6 | pRS316 | Auto-replicating | *CST6; URA3* | This work |
| pScNCE103-GFP | pRS316 | Auto-replicating | Sc*NCE103-GFP*; *URA3* | This work |
| pScNCE103-GFP-MUT | pNCE103-GFP | Auto-replicating | Sc*NCE103-GFP*; *URA3* | This work |
| pUG6 |  |  | *loxP-kanMX-loxP* | Guldener et al., 1996 |
| pUG72 |  |  | *loxP-URA3-loxP* | Guldener et al., 2002 |
| pTEF-GFP | pYM-N21 | Auto-replicating | *GFP* | This work |
