# Supplementary material for: The bZIP Transcription Factor Rca1p Is a Central Regulator of a Novel CO2 Sensing Pathway in Yeast
Source: PLoS Pathog. 2012 Jan 12;8(1):e1002485. doi: 10.1371/journal.ppat.1002485 (PMC3257301; doi:10.1371/journal.ppat.1002485)
Supplement: Table S4 — Strains used and constructed in this study. (DOCX) [file ppat.1002485.s013.docx]

| **Name** | **Species** | **Parental strain** | **Genotype** | **Reference** |
| --- | --- | --- | --- | --- |
| CAI4 | *C. albicans* |  | *ura3::imm434/ura3::imm434* | Fonzi and Irwin, 1993 |
| CAI4+pSM2 | *C. albicans* | CAI4 | *ura3::imm434/ura3::imm434*-(pSM2) | Hall et al., 2010 |
| TK1 | *C. albicans* | CAI4 | *nce103::hisG/nce103::hisG ura3::imm434*/*ura3::imm434* | Klengel et al., 2005 |
| Ca*nce103Δ* | *C. albicans* | TK1 | *nce103::hisG/nce103::hisG ura3::imm434*/*ura3::imm434*-(pSM2) | This work |
| Ca*nce103Δ* + pCaNCE103 | *C. albicans* | TK1 | *nce103::hisG/nce103::hisG ura3::imm434*/*ura3::imm434*-(pMB5) | This work |
| *cyr1Δ* | *C. albicans* | CAI4 | *cyr1::hisG*/*cyr1::hisG ura3::imm434*/*ura3::imm434*-(pSM2) | This work |
| *rca1*/RCA1 | *C. albicans* | CAI4 | *rca1::hisG/RCA1 ura3::imm434*/*ura3::imm434* | This work |
| *rca1*/RCA1+RCA1 | *C. albicans* | *rca1*/RCA1 | *rca1::hisG/RCA1 ura3::imm434*/*ura3::imm434* -(pSM2-RCA1) | This work |
| *rca1*/RCA1+RCA1-HA3 | *C. albicans* | *rca1*/RCA1 | *rca1::hisG/RCA1 ura3::imm434*/*ura3::imm434* -(pSM2-RCA1-HA3) | This work |
| *rca1Δ ura3Δ* | *C. albicans* | *rca1*/RCA1 | *rca1::hisG/rca1::hisG ura3::imm434*/*ura3::imm434* | This work |
| *rca1Δ* | *C. albicans* | *rca1Δ ura3Δ* | *rca1::hisG/rca1::hisG ura3::imm434*/*ura3::imm434* -(pSM2) | This work |
| *rca1Δ*+RCA1 | *C. albicans* | *rca1Δ ura3Δ* | *rca1::hisG/rca1::hisG ura3::imm434*/*ura3::imm434* -(pSM2-RCA1) | This work |
| *rca1Δ*+RCA1-HA3 | *C. albicans* | *rca1Δ ura3Δ* | *rca1::hisG/rca1::hisG ura3::imm434*/*ura3::imm434* -(pSM2-RCA1-HA3) | This work |
| *rca1Δ*+RCA1-S124A | *C. albicans* | *rca1Δ ura3Δ* | *rca1::hisG/rca1::hisG ura3::imm434*/*ura3::imm434* -(pSM2-RCA1-S124A) | This work |
| *rca1Δ*+RCA1-S126A | *C. albicans* | *rca1Δ ura3Δ* | *rca1::hisG/rca1::hisG ura3::imm434*/*ura3::imm434* -(pSM2-RCA1-S126A) | This work |
| *rca1Δ*+RCA1-S222G | *C. albicans* | *rca1Δ ura3Δ* | *rca1::hisG/rca1::hisG ura3::imm434*/*ura3::imm434* -(pSM2-RCA1-S222G) | This work |
| BY4741 | *S. cerevisiae* |  | *MATa his3Δ leu2Δ met15Δ ura3Δ* | This work |
| Sc*nce103Δ* | *S. cerevisiae* | BY4741 | *MATa his3Δ leu2Δ met15Δ ura3Δ nce103Δ* | This work |
| Sc*nce103Δ* + ScNCE103-GFP | *S. cerevisiae* | Sc*nce103Δ* | *MATa his3Δ leu2Δ met15Δ ura3Δ nce103Δ* + pNCE103-GFP | This work |
| Sc*nce103Δ* + ScNCE103-GFP-MUT | *S. cerevisiae* | Sc*nce103Δ* | *MATa his3Δ leu2Δ met15Δ ura3Δ nce103Δ* + pNCE103-GFP-MUT | This work |
| ScNCE103-GFP | *S. cerevisiae* | BY4741 | *MATα his3Δ1 leu2Δ lys2Δ ura3Δ NCE103-GFP:HIS3MX6* | Invitrogen |
| ScNCE103-GFP + pRS316 | *S. cerevisiae* | ScNCE103-GFP | *MATα his3Δ1 leu2Δ lys2Δ ura3Δ NCE103-GFP:HIS3MX6* + pRS316 | This work |
| ScNCE103-GFP*+cst6Δ* | *S. cerevisiae* | NCE103-GFP | *MATα his3Δ leu2Δ lys2Δ ura3Δ cst6::URA3 NCE103-GFP:HIS3MX6* | This work |
| ScNCE103-GFP*+cst6ΔKan* | *S. cerevisiae* | ScNCE103-GFP | *MATα his3Δ leu2Δ lys2Δ ura3Δ cst6::KAN NCE103-GFP:HIS3MX6* | This work |
| ScNCE103-GFP*+cst6Δ* + pRS316 | *S. cerevisiae* | ScNCE103-GFP*+cst6ΔKan* | *MATα his3Δ leu2Δ lys2Δ ura3Δ cst6::KAN NCE103-GFP:HIS3MX6* + pRS316 | This work |
| ScNCE103-GFP*+cst6Δ* + pRS316-CST6 | *S. cerevisiae* | ScNCE103-GFP*+cst6ΔKan* | *MATα his3Δ leu2Δ lys2Δ ura3Δ cst6::KAN NCE103-GFP:HIS3MX6* + pRS316-CST6 | This work |
| BY4741+pTEF-GFP | *S. cerevisiae* | BY4741 | *MATa his3Δ leu2Δ met15Δ ura3Δ* + pTET-GFP | This work |
| BL21(DE3) | *E. coli* |  | F– *omp*T *hsd*SB(rB–, mB–) *gal dcm* (DE3) | Invitrogen |
| BL21(DE3)+pGEX-6P-2-NCE103 | *E. coli* | BL21(DE3) | F– *omp*T *hsd*SB(rB–, mB–) *gal dcm* (DE3) + pGEX-6P-2-NCE103 | This work |
