# Supplementary material for: The bZIP Transcription Factor Rca1p Is a Central Regulator of a Novel CO2 Sensing Pathway in Yeast
Source: PLoS Pathog. 2012 Jan 12;8(1):e1002485. doi: 10.1371/journal.ppat.1002485 (PMC3257301; doi:10.1371/journal.ppat.1002485)
Supplement: Table S5 — Primers used in this study. (DOCX) [file ppat.1002485.s014.docx]

| **Name** | **Sequences (orientated 5' to 3')** |
| --- | --- |
| RCA1-F-SacI | GGGGGGGAGCTCGAGTACATCCACCCGTGCGG |
| RCA1-R-BglII | GGGGGGAGATCTCTTCTTCGACGTGGAACCCC |
| RCA1-F-BamHI | GGGGGGGGATCCCTGAGTTGGAGTATACGGTG |
| RCA1-R-HindIII | GGGGGGAAGCTTCCATTAGAGCACTGGCCATC |
| pMB5-F | GGGGGGTCTAGACGGATAAATCCTACTGCCTAT |
| pMB5-R | GGGGGGGGATCCTCAATGAGGGTTATATTCTTCT |
| Orf19.6102-F2 | GGGGGGAGATCTGGTTGAGTCGGTTGTTTCTG |
| Orf19.6102-R2 | GGGGGGGCGGCCGCGGCCATCACTCAGAGCGGTC |
| Orf19.6102(HA) | TATAGGTACCTTAGCGGCCGCCAATAAATTGCTTCAACCTGTCGG |
| Orf19.6102-HA-R2 | GGGGGGAGATCTTTAGCGGCCGCACTGAGCAG |
| S124A-F | GAAGAATAGCGATATCGAATGGTC |
| S124A-R | GACCATTCGATATCGCTATTCTTC |
| S126A-F | GAAGAATATCGATAGCGAATGGTC |
| S126A-R | GACCATTCGCTATCGATATTCTTC |
| S222G-F | ATTGCTGCCGGCAAGTGTAGG |
| S222G-R | CCTACACTTGCCGGCACGAAT |
| ORF19-6102ATG | GTCGATTATGGGCCATAATGATTTGTGCGAAAACAACGGGATACCAAAGAGTTTTATTCCACGTTCGTGCTCGATGATAAAGTCGAACTGGTAAGGGTGGGAAGCTTCGTACGCTGCAGGTC |
| ORF19-6102STOP | AAAACCCACTACACTGCTGAAACTCCTGTTGCTGGTGTTTCGCGTATCATTCAAACGTCTATTGGCCCGGGGCCTCAAGAAACTAGTGAATCATTAACTATCTGATATCATCGATGAATTCGAG |
| Nce.Ko.Kan-F | CTACAGCTAAGACTACAAATTTCAATTATTACACATCAGACAGCTGAAGCTTCGTACGC |
| Nce.Ko.Kan-R | CAATGAATATTATATAAGTATATCGGTGAGGCTAAAACTAGCATAGGCCACTAGTGGATCTG |
| ScCST6.Ko.Kan-F | CTAAAGTAGTAAAGAAAAAAGTATAAGCCCACACCTTTTTGGTAGGATACAGCTGAAGCTTCGTACGC |
| ScCST6.Ko.Kan-R | GTCCTTATCATTCTTGAATGAAACACCGTTGTGCTCACCAAAACTTTGGCATAGGCCACTAGTGGATCTG |
| ScNCE-1 | GGGGGGGCGGCCGCGCTAAATAACAATACCGAGC |
| ScNCE-end | GGGGGGGGATCCGAGTGGGGCTAAATATCCCC |
| CaNCE-FBamGEX | GGATCCGGTAGAGAAAATATTTTGAAATATCAATTGG |
| NCE-BR | CTTGCATATTATGCAATTGGACGTTAG |
| RCA1-F-SacI | GGGGGGGAGCTCGAGTACATCCACCCGTGCGG |
| RCA1-R-BglII | GGGGGGAGATCTCTTCTTCGACGTGGAACCCC |
| RCA1-F-BamHI | GGGGGGGGATCCCTGAGTTGGAGTATACGGTG |
| RCA1-R-HindIII | GGGGGGAAGCTTCCATTAGAGCACTGGCCATC |
| pMB5-F | GGGGGGTCTAGACGGATAAATCCTACTGCCTAT |
| pMB5-R | GGGGGGGGATCCTCAATGAGGGTTATATTCTTCT |
| Orf19.6102-F2 | GGGGGGAGATCTGGTTGAGTCGGTTGTTTCTG |
| Orf19.6102-R2 | GGGGGGGCGGCCGCGGCCATCACTCAGAGCGGTC |
| Orf19.6102(HA) | TATAGGTACCTTAGCGGCCGCCAATAAATTGCTTCAACCTGTCGG |
| Orf19.6102-HA-R2 | GGGGGGAGATCTTTAGCGGCCGCACTGAGCAG |
| S124A-F | GAAGAATAGCGATATCGAATGGTC |
| S124A-R | GACCATTCGATATCGCTATTCTTC |
| S126A-F | GAAGAATATCGATAGCGAATGGTC |
| S126A-R | GACCATTCGCTATCGATATTCTTC |
| S222G-F | ATTGCTGCCGGCAAGTGTAGG |
| S222G-R | CCTACACTTGCCGGCACGAAT |
| CaNCE-FBamGEX | GGATCCGGTAGAGAAAATATTTTGAAATATCAATTGG |
| NCE-BR | CTTGCATATTATGCAATTGGACGTTAG |
| Nce.Ko.Kan-F | CTACAGCTAAGACTACAAATTTCAATTATTACACATCAGACAGCTGAAGCTTCGTACGC |
| Nce.Ko.Kan-R | CAATGAATATTATATAAGTATATCGGTGAGGCTAAAACTAGCATAGGCCACTAGTGGATCTG |
| ScCST6.Ko.Kan-F | CTAAAGTAGTAAAGAAAAAAGTATAAGCCCACACCTTTTTGGTAGGATACAGCTGAAGCTTCGTACGC |
| ScCST6.Ko.Kan-R | GTCCTTATCATTCTTGAATGAAACACCGTTGTGCTCACCAAAACTTTGGCATAGGCCACTAGTGGATCTG |
| ScNCE-1 | GGGGGGGCGGCCGCGCTAAATAACAATACCGAGC |
| ScNCE-end | GGGGGGGGATCCGAGTGGGGCTAAATATCCCC |
| NCE103-Verif-F | GGGGGGGCGGCCGCGTAACGTAAGTCACGTGCTTTC |
| CST6-Verif-F | CGTTGTTGTGGCTGTCGCTG |
| CST6-Verif-R | CTAAGGAAGCGATGGGACAC |
| CST6-F | GGGGGGCGGCCGCGATCAATTGTATTATCAATG |
| CST6-R | GGGGGGAAGCTTGACCAGCTTGTCAAGATCGTC |
| FwGP | ATCAAAGAAGGTTAATGTGGCTGTGGTTTCAGGGTCCATACGTACGCTGCAGGTCGAC |
| RvGP | TTTTTTTTTCGTCATTATAGAAATCATTACGACCGAGATTCATCGATGAATTCTCTGTCG |
| **Quantitative Real-Time PCR** | |
| CaACT1-RT-F | CCTACGTGTACTTGTGCAAGGCAA |
| CaACT1-RT-R | TAGTTGTGTGCACTGAGCGTCGAA |
| CaORF19.6103-RT-F | CACGGGTGGATGTACTCAAA |
| CaORF19.6103-RT-R | GGGAGCATTTCTTCCTTAGTC |
| CaMVD-RT-F | GTTTCAGCAATCGCAAAGTT |
| CaMVD-RT-R | TCCGTCAGGTAAAGTACCCA |
| CaRCA1-RT-F | TTGCCAAGATGGAGAAAGAG |
| CaRCA1-RT-R | TCGGTGTTGCCATCTTCTAT |
| CaCHT2-RT-F | AATGTGTTGCCACTCCAGTT |
| CaCHT2-RT-R | CGGTGCATACAACAGTTTGA |
| CaOCH1-RT-F | ATGTCAATCAAATGGGTGCT |
| CaOCH1-RT-R | GGCATACCATCATCTTTCCA |
| Ca-ChIP-NCE103-F | CCATCACAGTTTCGGATAAATC |
| Ca-ChIP-NCE103-R | GTATTGACGTAAGTAGAGTCAC |
| Ca-ChIP-ACT1-F | CTCACCAAGATTTATTGCCAAC |
| Ca-ChIP-ACT1-F | CACCCTACCCATTTGTCATATT |
| CaNCE103-RT-F | AGGGTGTAGTGATTCAAGAGCAGG |
| CaNCE103-RT-R | GATGCCCAAATACCACCACAATCAG |
| ScNCE103-RT-F | GCCACTTTAGAGTTTGCCATT |
| ScNCE103-RT-R | TTTGGTAAGGCTTCCCTTTG |
| ScACT1-RT-F | TGCCGGTATTGACCAAACTA |
| ScACT1-RT-R | ATACCTGGGAACATGGTGGT |
| ScCst6 RT-F | ACCTATGACGAATCCACCAA |
| ScCst6 RT-R | GCTGGCTTATTTGACCGTTA |
